# Canopy structural diversity mediates the effect of climate on primary productivity in forests

**DOI:** 10.1038/s41467-026-75043-3

**Published:** 2026-07-11

**Authors:** Xiaoxia Yi, Grégoire Vincent, Tianyu Hu, Jianli Liu, James A. Lutz, Haibao Ren, Lei Chen, David F. R. P. Burslem, Joseph D. Birch, Min Cao, James W. Dalling, David A. Coomes, Géraldine Derroire, Hu Du, Shuai Fang, Guangze Jin, Jinbo Li, Fei Lin, Hongwei Ni, Michael J. O’Brien, Guochun Shen, Xihua Wang, Xingyu Wang, Jie Yang, Fuping Zeng, Daoguang Zhu, Yan Zhu, Yanjun Su, Qinghua Guo, Keping Ma, Xiangcheng Mi

**Affiliations:** 1 Zhejiang Qianjiangyuan Forest Biodiversity National Observation and Research Station, Key Laboratory of Vegetation and Environmental Change, Institute of Botany, Chinese Academy of Sciences, Beijing, China; 2 China National Botanical Garden, Beijing, China; 3 University of the Chinese Academy of Sciences, Beijing, China; 4 AMAP, Univ. Montpellier, CIRAD, CNRS, INRAE, IRD, Montpellier, France; 5 College of Geography and Geomatics, Xuchang University, Xuchang, China; 6 Wildland Resources Department, Utah State University, 5230 Old Main Hill, Logan, UT USA; 7 The Ecology Center, Utah State University, 5230 Old Main Hill, Logan, UT USA; 8 School of Biological Sciences, University of Aberdeen, Cruickshank Building, St Machar Drive, Aberdeen, UK; 9 CAS Key Laboratory of Tropical Forest Ecology, Xishuangbanna Tropical Botanical Garden, Chinese Academy of Sciences, Kunming, China; 10 Center of Conservation Biology, Core Botanical Gardens, Chinese Academy of Sciences, Wuhan, China; 11 Department of Plant Biology, University of Illinois at Urbana-Champaign, Urbana, IL USA; 12 Smithsonian Tropical Research Institute, Apartado, Balboa, Panama; 13 University of Cambridge, Cambridge, UK; 14 Cirad, UMR EcoFoG (AgroParistech, CNRS, INRAE, Université des Antilles, Université de la Guyane), Campus Agronomique, Kourou, French Guiana; 15 Cirad, UPR Forêts et Sociétés, University of Montpellier, Montpellier, France; 16 University of Brasilia, Department of Forestry, Campus Darcy Ribeiro, Brasília, Brazil; 17 Key Laboratory of Agro-ecological Processes in Subtropical Region, Institute of Subtropical Agriculture, Chinese Academy of Sciences, Changsha, Hunan China; 18 CAS Key Laboratory of Forest Ecology and Silviculture, Institute of Applied Ecology, Chinese Academy of Sciences, Shenyang, China; 19 Key Laboratory of Terrestrial Ecosystem Carbon Neutrality, Shenyang, Liaoning Province China; 20 Center for Ecological Research, Northeast Forestry University, Harbin, China; 21 Institute of Natural Resources and Ecology, Heilongjiang Academy of Sciences, Harbin, China; 22 Heilongjiang Academy of Forestry, Harbin, China; 23 Estación Experimental de Zonas Áridas, Consejo Superior de Investigaciones Científicas, Almería, Spain; 24 Zhejiang Tiantong Forest Ecosystem National Observation and Research Station, School of Ecological and Environmental Sciences, East China Normal University, Shanghai, China; 25 Institute of Ecology, College of Urban and Environmental Science, Peking University, Beijing, China

**Keywords:** Community ecology, Ecosystem ecology, Forest ecology

## Abstract

Canopy structural diversity varies systematically with climate and species diversity across boreal, temperate, and tropical biomes. Yet, how this latitudinal variation affects forest productivity—particularly the role of structural diversity in mediating effects of climate on productivity across biomes—remains unresolved. By synthesizing airborne laser scanning data and ground-based forest inventories spanning boreal to tropical forests, we show that beyond its direct effects on forest productivity, climate influences productivity indirectly by regulating canopy structural diversity—a significant, yet previously underappreciated mechanism whose precise magnitude is challenging to isolate. Notably, we observe a pronounced latitudinal congruence between hotspots of structural diversity and productivity. This positions structural diversity not only as a complementary indicator of productivity but also as a critical mediator of the influence of climate and species diversity, essential for improved forecasting.

## Abstract (web summary)

How climate regulates forest productivity across biomes remains unclear. This study shows that climate not only directly affects productivity but also indirectly influences it by regulating canopy structural diversity.

## Introduction

Forest canopies—the collective layer of tree crowns in a stand—capture up to 98% of incoming solar energy in dense forests^1,2^, highlighting the role of canopy structural diversity in driving ecosystem energy capture. Canopy structural diversity—the three-dimensional heterogeneity in volume and arrangement of above-ground vegetation (e.g., leaves and branches)—is an emergent property of canopy height, canopy cover, and vertical complexity (i.e., canopy depth and vertical leaf area distribution)^3–5^. These structural components are expected to influence productivity through three mechanistic pathways: (1) space filling, where greater horizontal cover and vertical stratification increase the total light-intercepting leaf area; (2) light-environment partitioning, where heterogeneous arrangements of leaf area create vertical light gradients within canopies; and (3) vertical niche differentiation, where coexisting species with divergent leaf and crown traits exploit these light gradients more efficiently^5–9^. Consequently, structural diversity is hypothesized to govern not only the total amount of light intercepted but also how light is partitioned and utilized within canopies, thereby directly coupling structural diversity to stand-scale productivity^10^. The strength of this relationship, however, may be modulated by climate via two pathways: directly, through physiological constraints imposed by water stress and temperature extremes on individual tree growth^11^, and indirectly, through climate-driven changes in structural diversity^12,13^. These structural changes may modify the internal light environment, potentially influencing overall light interception and use efficiency by facilitating exploitation among coexisting species with divergent leaf traits or by limiting it in species with less differentiated leaf traits^3,5^. Resolving the mechanistic link between these climate-driven shifts in structural diversity and observed productivity patterns across spatial gradients thus requires disentangling the dual roles of climate: its direct physiological constraints on growth versus its indirect effects on productivity mediated by structural diversity.

**Fig. 1:**
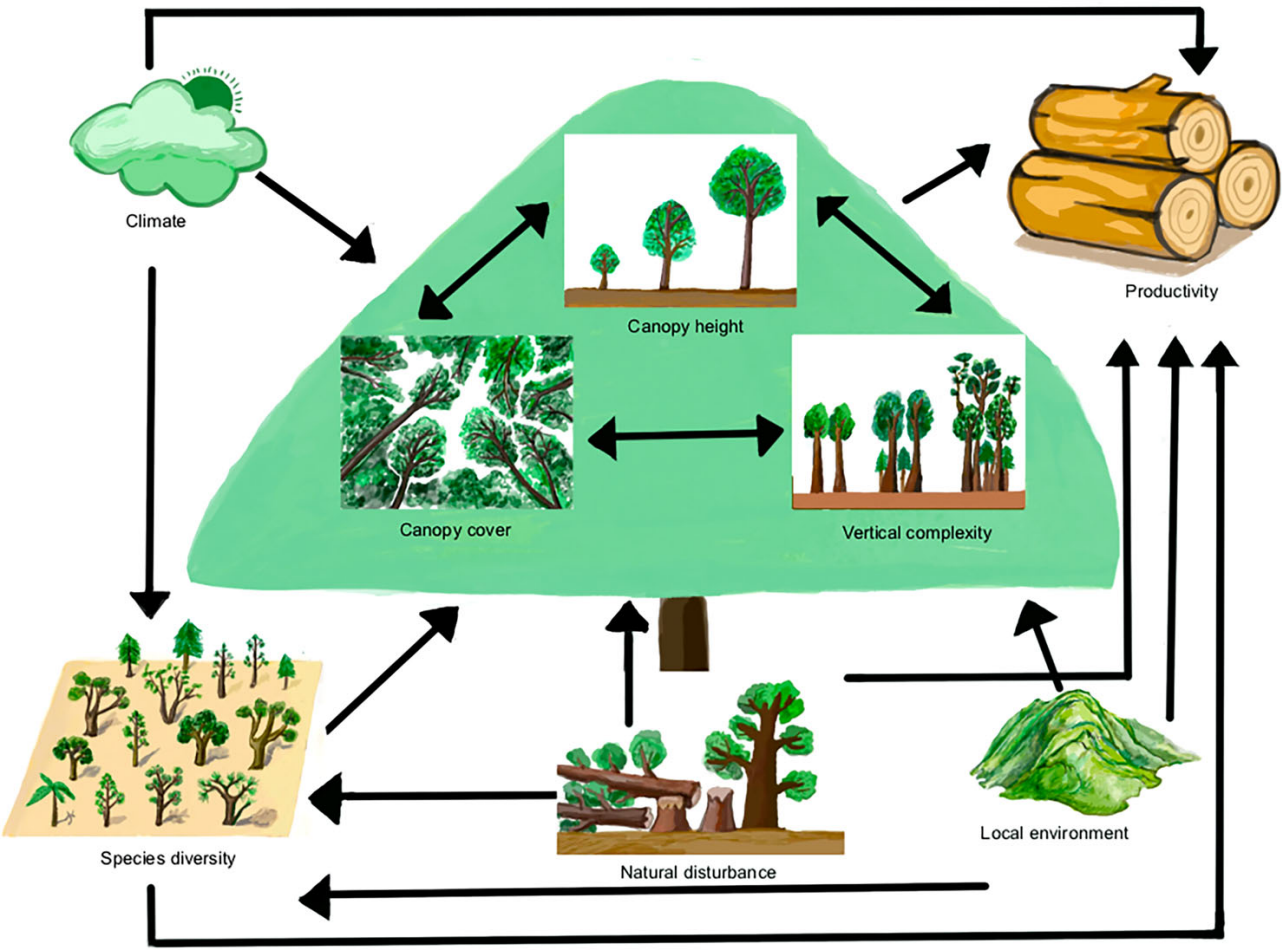
Conceptual illustration of direct and indirect drivers of forest productivity. Piecewise structural equation modeling (SEM) accounting for direct and indirect effects of climate, species diversity (quantified as species richness), components of canopy structural diversity, natural disturbance and local environment (e.g., topography) on forest productivity. This conceptual illustration for drivers of forest productivity was created by Xiangtao Su.

**Fig. 2:**
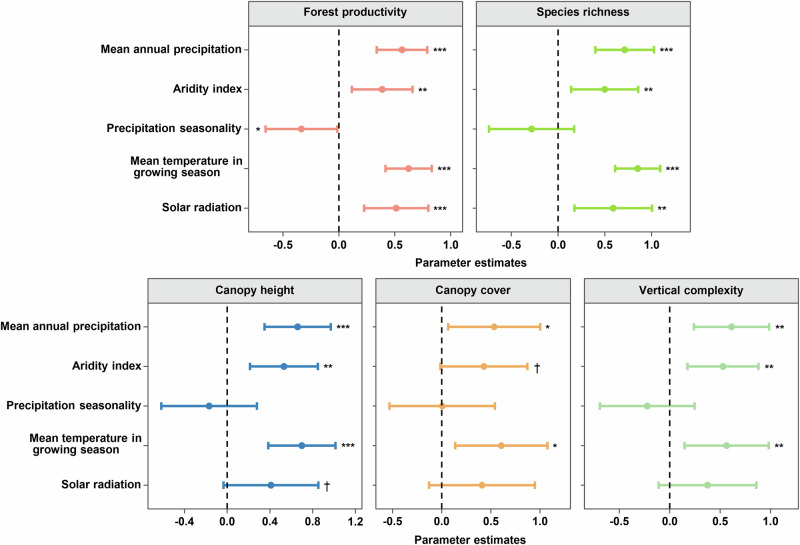
Effects of individual climate variables on forest productivity, species richness and components of canopy structural diversity at 50 m × 50 m scale. Points show the mean effect size and error bars represent 95% confidence intervals (*n* = 1568 quadrats with 27-200 quadrats per plot). An error bar not overlapping with zero indicates a significant effect. The effect size and *p* values were calculated based on linear mixed-effects models with plot-level random effects (see Supplementary Table 8 for full results). Significance levels: †*p* < 0.10, *p  <  0.05, **p  <  0.01, ***p  <  0.001. Statistical tests were two-sided, and no adjustments were made for multiple comparisons. Source Data is provided as a Source Data file.

The direct effect of climate through physiological constraints manifests in broad spatial patterns of forest productivity. Forest productivity tends to increase with temperature in cold regions^14^ and with precipitation in dry forests^15^. In parallel, climate exerts potent indirect effects by shaping canopy structural diversity along a latitudinal gradient. The hydraulic limitation hypothesis proposes that hydraulic stress in drier climates limits tree height growth^16,17^, whereas higher water availability in wetter climates facilitates multi-layered canopies with larger trees^12,18,19^, enhancing spatial occupancy and vertical stratification. Warmer and wetter climates further support species-rich communities with diverse functional strategies^20^ that potentially enhance crown spatial complementarity and niche differentiation. The synergy between climate-driven shifts in structural diversity and direct physiological effects intensifies toward the tropics, thereby strengthening the relationship between structural diversity and forest productivity across latitudinal gradients^6,7,12,21,22^. Collectively, this line of reasoning supports two predictions: (1) an indirect role of climate in shaping forest productivity via structural diversity, along with direct physiological effects (e.g., photosynthesis, respiration) (Fig. 1), and (2) a positive relationship between canopy structural diversity and forest productivity that correlates with latitude. These predictions remain empirically unvalidated across biome-scale gradients, constrained by technical limitations in deploying high-resolution terrestrial/airborne LiDAR (light detection and ranging) for cross-biome analyses. Previous studies have focused on temperate forests^6,23–25^ or relied on spaceborne LiDAR for global assessments with coarse resolution limits^22^. Consequently, the role of canopy structural shifts in mediating climate-associated productivity patterns remains unclear across biomes.

Here we test the two predictions empirically by examining how climate, species diversity and canopy structural diversity shape the latitudinal gradient in forest productivity. To do so, we employ structural equation modeling (SEM), a framework that allows the direct effects of climate and diversity on productivity to be disentangled from their indirect effects mediated through canopy structural diversity, while accounting for local environmental variation and natural disturbance (Fig. 1). Accordingly, we compile inter-calibrated airborne laser scanning and ground-based forest inventories from 16 large stem-mapped forest dynamics plots (13.64-50 ha) spanning boreal to tropical forests (Supplementary Fig. 1, Supplementary Table 1). These plots, drawn from the ForestGEO and the Chinese Forest Biodiversity Monitoring networks^26,27^, capture latitudinal gradients in climate and biodiversity in natural forests. Climate variables (e.g., mean annual temperature and precipitation) are extracted from WorldClim (1-km² resolution) using plot centroid coordinates^28^. At each plot, we quantify components of canopy structural diversity—specifically canopy height, canopy cover, and vertical complexity—using LiDAR derived metrics for non-overlapping 1568 50 m × 50 m quadrats and 385 100 m × 100 m quadrats (Supplementary Table 1). These components of structural diversity reflect spatial occupancy, horizontal heterogeneity and crown complementarity^6,7^. Species diversity and forest productivity are also estimated at matching spatial scales. We quantify species diversity as species richness per quadrat, since richness can influence productivity via niche complementarity and sampling effects^29^. Forest productivity is quantified as above-ground net primary productivity, calculated as annual woody biomass increments using allometric equations and repeated census data^30^. Topographic variables (e.g., elevation and slope) and quadrat-scale natural disturbance (i.e., events caused by non-anthropogenic agents—such as fire, wind, or pathogens—that significantly alter forest structure and functions, initiating succession^31^, see more details in ‘Methods’) are included as covariates to account for local environmental conditions and natural disturbance. Because results for the 100 m × 100 m quadrat size are similar to those for the 50 m × 50 m size across all analyses, we present results for the 50 m × 50 m quadrats only. We find that the indirect effect of climate on productivity, mediated through canopy structural diversity, represents a previously overlooked pathway that complements the well-known direct physiological constraints of climate. We further observe a spatial association between hotspots of structural diversity and forest productivity. These insights highlight the importance of integrating climate-driven changes in structural diversity into ecosystem models for understanding and forecasting ecosystem responses to climate change.

## Results

### Climate effects on productivity, species diversity and canopy structure

Forest productivity increased with water availability (mean annual precipitation and aridity), temperature (mean temperature in the growing season) and light availability (solar radiation) (Fig. 2). Temperature and precipitation seasonality—the model’s fixed effects, or the specific explanatory variables under evaluation—explained 42% of the variation in productivity across plots (Supplementary Table 2, marginal *R*^2^ [*R*^2^_M_] = 0.42). The full model, which also accounted for random effect (i.e., the variation attributable to the grouping structure of the data, such as quadrats within plots), explained 55% of this variation (Supplementary Table 2, conditional *R*^2^ [*R*^2^_c_] = 0.55). Variation in species richness was closely correlated with most climate variables other than precipitation seasonality (Fig. 2, Supplementary Table 2, R2M = 0.69).

We found strong correlations between components of structural diversity measured from LiDAR data and several climate variables (e.g., water availability and temperature) (Fig. 2, Supplementary Table 2, R2M = 0.22-0.49). However, we found no relationships with solar radiation and precipitation seasonality (Fig. 2).

### Bivariate relationships between productivity and species diversity, natural disturbance and canopy structure

**Fig. 3:**
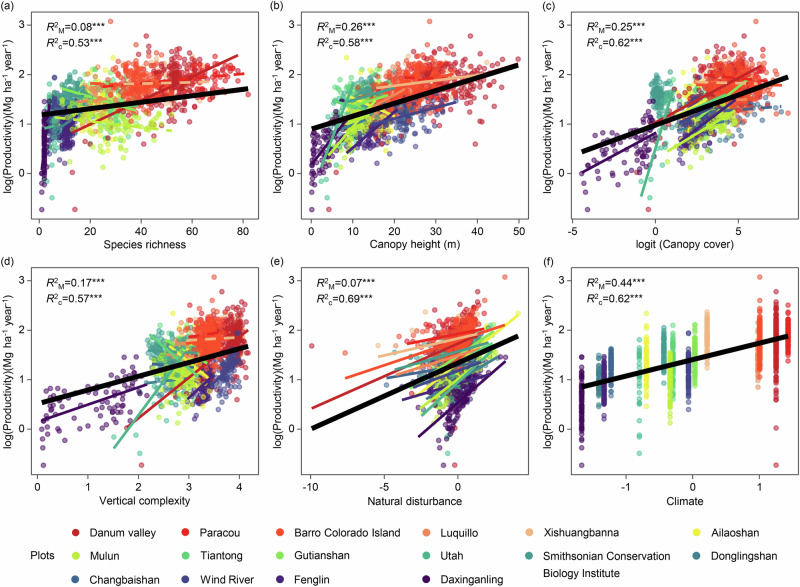
Relationships between forest productivity and (**a**) species richness, (**b**) canopy height, (**c**) canopy cover, (**d**) vertical complexity, (**e**) natural disturbance, (**f**) climate at 50 m × 50 m scale. (*n* = 1568 quadrats). Climate was represented by the scores of plots in the first axis of principal component analysis of five climatic variables used in this study (Supplementary Table 6). Natural disturbance history was represented by net annual biomass change, standardized to unit variance within its respective plot (see details in “Methods” section). Colored points correspond to values of quadrats in each forest plot, and the colored lines indicate the fitted generalized least squares models with the lowest AIC scores of each plot. Solid lines represent significant relationships (*p* < 0.10) and dashed lines represent non-significant relationships (*p* ≥ 0.10). The bold black lines depict the fitted linear mixed-effects models across forest biomes (see Supplementary Table 3 for full results). The marginal *R*^2^ (*R*^2^_M_) and conditional *R*^2^ (*R*^2^_C_) are used to represent the proportion of variance explained by each predictor with and without accounting for the random effect of plot identity (see details in Source Data 1). Levels of significance: * *p* < 0.05, *** *p* < 0.001. Statistical tests were two-sided, and no adjustments were made for multiple comparisons.

Across plots, we found that forest productivity was positively correlated with species richness, climate, natural disturbance, canopy height, canopy cover and vertical complexity. Notably, the three components of canopy structural diversity explained more variation in productivity (canopy height [*R*^2^_M_ = 0.26], canopy cover [*R*^2^_M_ = 0.25], and vertical complexity [*R*^2^_M_ = 0.17]) than species richness (*R*^2^_M_ = 0.08) alone. However, within individual plots, the relationships between productivity and both components of structural diversity and species richness were variable, including negative correlations, though the overall trend was positive. In contrast, natural disturbance exhibited a consistent positive correlation with productivity (*R*^2^_M_ = 0.07, Fig. 3, Supplementary Table 3, Supplementary Data 1).

### Direct and indirect effects of species richness, natural disturbance, structural diversity and climate on productivity

**Fig. 4:**
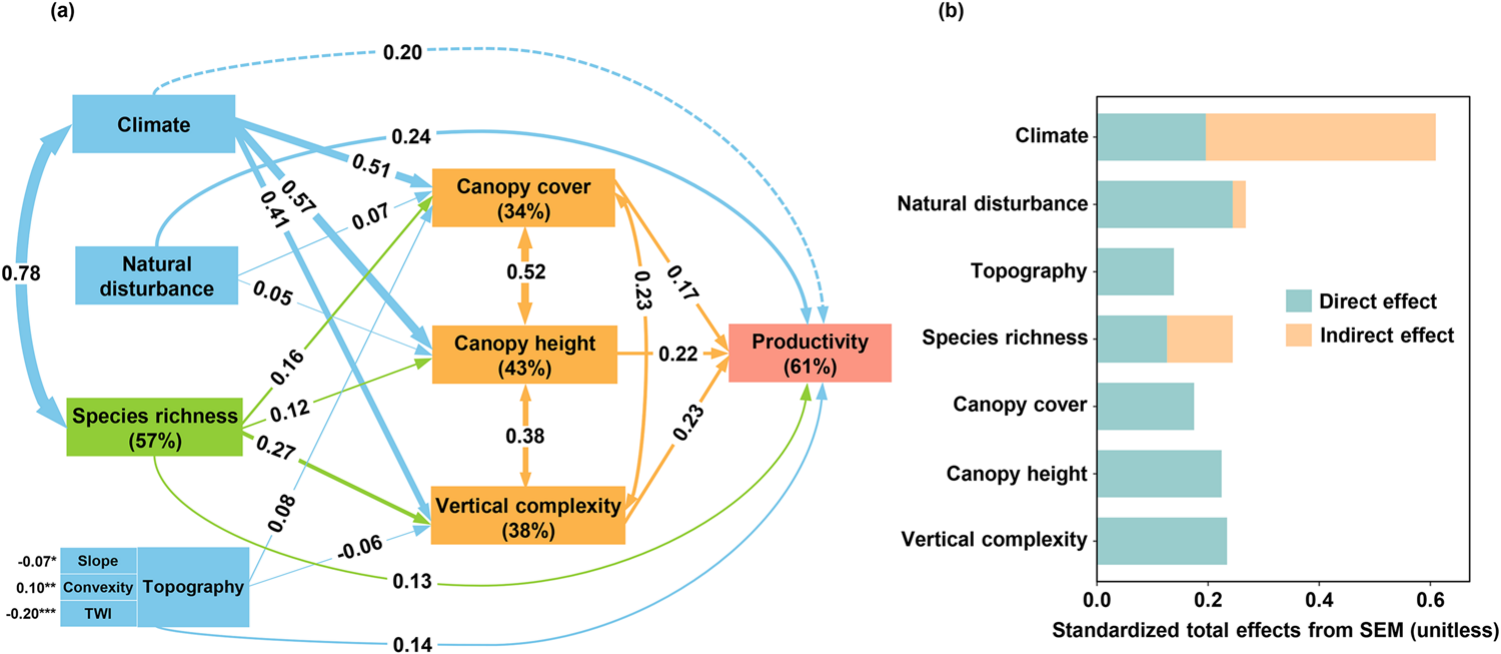
Piecewise structural equation models (SEM) of species richness, components of canopy structural diversity, natural disturbance, topography and climate as predictors of forest productivity at 50 m × 50 m scale. (*n* = 1568 quadrats, Fisher’s C = 5.02, *p* = 0.76, *df* = 8, AIC = 8804.87). Direct and indirect effects (**a**) and the standardized total effects (**b**) of different factors on productivity are shown. The numbers on the arrows represent the standardized path coefficients with arrow width corresponding to the strength of these coefficients (see Supplementary Table 9 for full results). Marginal *R*^2^ values in brackets represent the percentage of variance explained by all predictors after accounting for random effect of ‘plot’. Solid arrows represent significant (*p* < 0.05) paths, while dashed arrows represent non-significant paths (*p* ≥ 0.05). Climate is the first principal component of a principal component analysis with six climatic variables, capturing 70.3% of the total variance in the climatic data (see Supplementary Table 6). Key topographical variables were linearly combined into a composite variable, ‘Topography’, and the numbers adjacent to each variable represent their weights used in this linear combination. Natural disturbance history was proxied using net annual biomass change. TWI stands for the topographic wetness index of a quadrat (see details in “Methods” section). Statistical tests were two-sided, and no adjustments were made for multiple comparisons. Source Data are provided as a Source Data file.

Climate and species richness positively regulated productivity both directly and indirectly through their positive effects on components of structural diversity (Fig. 4a, b). Climate had a stronger positive effect on productivity than species richness, natural disturbance or local topography alone. In addition, the combined influence of components of structural diversity collectively had a greater positive effect on productivity than either climate or species richness alone (Fig. 4b). Structural diversity remained positively correlated with productivity, even after controlling for climate, species richness, local topography and natural disturbance (Fig. 4a).

## Discussion

We confirmed the well-known direct effect of climate on forest productivity, widely described in current textbooks and attributed to direct influences of climate on key plant physiological processes, such as photosynthesis, respiration and hydrological dynamics^11^. This understanding has motivated extensive research on climate-driven productivity declines, particularly through mechanisms such as heat- and drought-induced growth suppression^32^ and the exceeding of species-specific thermal optima for photosynthesis^33^.

We were able to go beyond this direct effect, however, and found strong support for our first prediction that climate indirectly affects productivity across biomes by altering canopy structural diversity (e.g., crown complementarity, three-dimensional spatial heterogeneity). Structural diversity governs energy capture processes—such as space filling and light environment partitioning that enable vertical niche differentiation—that regulate how efficiently stands convert light into biomass^5,6,34^. While previous research has primarily emphasized the role of species diversity in driving productivity via canopy structural diversity at the community level^7–9,25^, our findings highlight that climate-driven variation in structural diversity itself may play an underappreciated role in modulating productivity across biomes. In our study, across broad latitudinal and climatic gradients, covariation between climate and species richness appears to shape structural diversity via physiological constraints on tree height, canopy cover, and vertical complexity^16,17^. In warm and wet tropical climates, milder hydraulic constraints facilitate taller and multilayered canopies with dense vertical species packing. This stratification is evident in canopy species adapted to high light environments (e.g., via lower specific leaf area) versus understory species specialized for shade (e.g., via higher specific leaf area)^35^. In contrast, colder or drier high-latitude climates impose stronger hydraulic and energy-balance limitations, favoring shorter, less stratified canopies with reduced light partitioning and niche differentiation. These climate-dependent shifts in structural diversity have direct consequences for stand-level productivity: more stratified canopies capture and utilize light more efficiently, whereas simplified canopies limit stand productivity^5,34^. A key implication is that structural diversity is missing from many dynamic global vegetation models, which characterize forest canopies using large-scale mean structural metrics (e.g., leaf area index and height) and therefore cannot capture structural diversity that governs those processes^36^. This omission may bias productivity estimates, underestimating productivity in structurally complex tropical forests while overestimating it in simpler high-latitude forests. Integrating climate-structural diversity-productivity feedbacks into next-generation vegetation models will improve ecosystem service projections and inform climate-adaptive forest management.

Estimating the indirect effect of climate on productivity is challenging because climate simultaneously exerts direct physiological influences on growth and shapes canopy architecture, creating strong dependence between the “treatment” and the mediator. This dependence can inflate the estimated mediation pathway in cross-sectional structural equation models. Nevertheless, while the *magnitude* of indirect effects may be overstated, the qualitative validation of structural mediation is supported by multiple lines of evidence: 1) experimental evidence showing structural complexity enhancements boost productivity independently of large-scale climate variation^7,37^; 2) consistent covariation of structural diversity and productivity along latitudinal climatic gradients in our analysis and prior work^12,22,23^. Future work integrating explicit mechanistic frameworks—such as process-based models or manipulative experiments—will be needed to disentangle direct climatic drivers from indirect structural diversity-mediated effects to strengthen causal inference.

Our results corroborated our second prediction that canopy structural diversity was positively correlated with productivity across forest biomes, even after controlling for climate, species diversity, natural disturbance and local topography. This finding aligns with global-scale analyses derived from spaceborne LiDAR^22^ and local-scale observations^6–8,34,38^, but conflicts with results from regional^39^ and other local-scale studies^40–43^. This discrepancy suggests that large-scale processes driving productivity—potentially linked to latitudinal gradients in diversity and evolutionary history—may override those operating locally and regionally. Large-scale patterns that could contribute include elevated functional diversity which may enhance canopy spatial complementarity among coexisting species^20^; deeper phylogenetic divergence among lineages fostering structural diversity^44^; and larger species pools increasing the probability of large-statured taxa^45^. We therefore recommend integrating spaceborne LiDAR (e.g., Global Ecosystem Dynamics Investigation, GEDI) to track climate-driven shifts in structural diversity, as a complementary indicator to assess the influence of climate change and species loss on forest productivity^46^.

Contrary to the positive relationship between structural diversity and productivity observed among plots, we found substantial variation in this relationship within plots across biomes. For instance, in temperate forests, the relationships are generally positive^6,23,24,38^, but can turn negative in stands with high structural diversity^40–42^. This context dependency likely reflects both intensified asymmetric competition in vertically complex forests—where canopy dominants suppress understory taxa through light limitation^47^— and interspecific differences in shade tolerance modulating vertical light and water partitioning^42^.

The primary limitation of our study, as discussed earlier, is the challenge of disentangling the direct and indirect effects of climate on productivity. This opens an opportunity for future research to develop frameworks that isolate direct climatic drivers from those mediated by structural diversity. Compounding this mechanistic challenge is a second, data-driven limitation: the omission of soil fertility due to a lack of consistent, plot-level data across our study area. Variation in soil properties (e.g., nutrient availability, organic matter) likely contributes to unexplained variance in our models, though some of its effects may be indirectly captured by climate and topography, with which soil development is closely linked^48^.

Our findings reveal that canopy structural diversity, shaped by both climate and species diversity, is a critical driver of forest productivity across biomes. We identify a consistent spatial correspondence between hotspots of canopy structural diversity and productivity, suggesting that monitoring canopy structural diversity could enhance assessments of forest productivity. While we do not directly assess climate change impacts, these spatial patterns offer insight into how structural shifts—potentially driven by climate change or biodiversity loss—could influence forest productivity. Finally, integrating climate-driven changes in structural diversity into ecosystem models may improve predictions of carbon sequestration, providing a more mechanistic understanding of forest responses to climate change.

## Methods

### Study sites

We included data from 16 forest dynamics plots (13.64–50 ha) spanning the world’s major forest biomes across latitudinal gradients in Asia and the Americas: boreal forests (2 plots), temperate seasonal forests (7), woodland/shrubland (2), tropical seasonal forest/savanna (4), and tropical rainforest (1) following the biome classification of Whittaker^49^ (Supplementary Fig. 1, Supplementary Table 1). Plots were drawn from the ForestGEO and the Chinese Forest Biodiversity Monitoring networks^26,27^ and represent natural forests with minimal anthropogenic disturbance. In 15 of the 16 plots, all woody stems with a diameter at breast height (DBH) ≥ 1 cm were tagged, spatially mapped and identified to species following a standardized protocol at 5-year intervals^50^. The Paracou plot constituted an exception to this protocol, with a census minimum DBH of 10 cm.

To temporally align forest census data with the LiDAR data (described below), we selected the two most recent censuses closest to the LiDAR survey dates (Supplementary Table 4). LiDAR acquisition occurred within the census interval for 9 plots, after the census ended for 6 plots (Luquillo, Gutianshan, Donglingshan, Tiantong, Fenglin and Daxinganling) and before the census began for 1 plot (Barro Colorado Island). Despite this variation, LiDAR data capture canopy structure typically within 0-5 years of the measured census period for all plots, supporting robust links between canopy structural diversity and forest productivity.

### Above-ground productivity and species diversity

Above-ground productivity for each quadrat was calculated as the sum of the above-ground biomass growth of surviving trees and the biomass of new recruits across two censuses, divided by the census interval in years^30^. Each forest plot was divided into non-overlapping 50 m × 50 m (49–200 per plot) and 100 m × 100 m (6–50 per plot) quadrats to balance the trade-off between minimizing the uncertainty in biomass estimation from border effects and maintaining sufficient sample size for robust cross-plot statistical analysis (Supplementary Table 1). This design mitigates the ~40% uncertainty from border effects at a 20 m × 20 m scale^51^ while ensuring sufficient sample size. Productivity was calculated from all trees with DBH ≥ 10 cm, which contribute more than 90% of stand-scale productivity^30^. For temperate North American plots (Utah, Smithsonian Conservation Biology Institute, Wind River), the aboveground biomass was estimated using the ‘allodb’ package, applying taxa-specific allometries calibrated with site-level climate data to mitigate local variability^52^. For temperate and subtropical Asian plots (Ailaoshan, Mulun, Gutianshan, Tiantong, Donglingshan, Changbaishan, Fenglin and Daxinganling), biomass estimates were derived from published species-specific allometric equations developed under similar bioclimatic conditions (see more details in Supplementary Data 2). For tropical plots (Danum Valley, Paracou, Barro Colorado Island, Luquillo and Xishuangbanna), aboveground biomass was calculated using the ‘BIOMASS’ package^53^. This package incorporates taxonomic identity and wood density from the Global Wood Density Database, and applies local height-diameter allometric corrections to account for site-specific differences^54^. For unidentified taxa or taxa lacking species- or genus-level wood density values, plot-level mean wood densities were applied.

Tree species richness was calculated by summing the number of species with at least one stem ≥ 10 cm DBH per 50 m × 50 m quadrat in the second census. To address undersampling bias in species-rich communities, tree species richness was also extrapolated using the ‘iNEXT’ R package^55^. Extrapolated estimates aligned closely with observed richness values (*r* = 0.89, *p* < 0.001).

### LiDAR data collection and processing

Small-footprint airborne LiDAR data were collected across all plots using sensors listed in Supplementary Table 4. To mitigate topographic effects, the raw point clouds were normalized to ground elevation (*z* = 0) via a triangular irregular network algorithm with bilinear interpolation^56^. To mitigate acquisition biases, pulse density was standardized to 5 pulses/m^2^ for most plots through random subsampling. Three plots (Danum Valley, Mulun, and the Smithsonian Conservation Biology Institute plot) retained their native pulse densities, which were close to this target (Supplementary Table 4)^56,57^.

For each 50 m × 50 m quadrat, we quantified canopy height, canopy cover and vertical complexity from first LiDAR returns, which are robust to variations in flight altitude and footprint size^58^. Canopy height was calculated as the average height of first returns (i.e., mean outer canopy height), assuming that first returns accurately represent the outer canopy surface due to limited penetration into vegetation layers^56^. Canopy cover was quantified as the proportion of the number of first returns above 2 m relative to the total number of first returns^56^, while vertical complexity was quantified as foliage height diversity, calculated by applying the Shannon-Wiener diversity function to the vertical distribution of first return heights^56^. Vertical complexity is expected to increase with greater canopy height extent and evenness in foliage layering^56^. All LiDAR metrics were calculated using the ‘lidR’ package^59^.

To assess metric robustness to LiDAR sensor differences, we analyzed airborne LiDAR data from a prior tropical forest study that employed three distinct sensors (used in 9 of our 16 plots)^60^. Canopy structural metrics—canopy height, cover and vertical complexity—exhibited high cross-sensor correlations (*r* > 0.92) for quadrats ≥ 25 m × 25 m (see Supplementary Methods). Although a full multi-sensor comparison was beyond the scope of this study, these results confirm that canopy structural metrics are comparable across plots when derived from quadrats ≥25 m × 25 m.

### Climatic and topographic data

To assess climatic controls on canopy structural diversity, species diversity, and forest productivity, we extracted the average values of climate variables for each plot from the WorldClim2 database (https://www.worldclim.org/)^28^. Using the central coordinates of each plot, we obtained 30 arcsecond (~1 km² resolution) raster data representing the 1971–2000 climatological baseline. We focused on six key climatic variables: (1) mean annual precipitation (mm), (2) precipitation seasonality (coefficient of variation of monthly precipitation), (3) solar radiation (kJ m² day^−1^), (4) mean temperature during the growing season (derived from the monthly average temperatures), (5) potential evapotranspiration (obtained from the Global Potential Evapotranspiration Database), and (6) the aridity index (the ratio of mean annual precipitation to mean annual potential evapotranspiration, obtained from the Global Aridity Index)^61^. Growing season duration was determined using biome-specific criteria: for boreal and temperate forests, we counted months with mean temperatures ≥ 5 °C, whereas for tropical/subtropical forests, we identified months where precipitation exceeded 50% of potential evapotranspiration (sensu FAO 1980).

We calculated six topographic variables to characterize the topography of each plot: the mean elevation, slope, convexity, aspect, topographic wetness index and altitude above channel for each quadrat. Mean elevation of a quadrat was the average elevation of its four corners; slope was derived from the average slope of the four triangular planes formed by connecting three corners of a quadrat; convexity was measured as the difference between the mean elevation of the focal quadrat and that of eight adjoining quadrats; aspect was defined as the direction of a slope (sine-transformed and cosine-transformed for analysis)^62^; the topographic wetness index was calculated as the ratio of the area upslope of each quadrat from any given point on the landscape to the local slope at that point; and altitude above channel was measured as the vertical distance above the nearest water channel in the valley^62^. All topographic variables were calculated using the ‘RSAGA’ package^63^ and the SAGA GIS software^64^.

### Effect of natural disturbance

Natural disturbances at stand scale (e.g., from fire, wind, or disease) can create canopy gaps, promoting the growth of smaller trees and shifting stand size structure towards younger, smaller individuals^31^. To evaluate these effects, we approximated disturbance history using three metrics: 1) mean individual basal area per quadrat as a proxy of development stage, 2) the skewness of DBH class distribution using 5 cm intervals, where a positive skew indicates a regenerating stand, and 3) net annual biomass change (calculated as biomass gain from growth and recruitment minus loss from mortality), where high net increases suggest recovery and net decreases signal recent disturbance. Biomass was estimated using the methods described in the “Above-ground productivity and species diversity” section. As these metrics were not directly comparable across plots, each was standardized to unit variance within its respective plot. While all three metrics showed significant but weak correlations with productivity (marginal *R*^2^ = 0.01–0.07, *p* < 0.001), we selected net annual biomass change as our representative measure because it directly quantifies the functional response of the ecosystem to disturbance by integrating the net effects of growth, recruitment, and mortality on carbon dynamics.

### Statistical analyses

We began by examining the effects of individual and combined climatic variables on productivity, species richness and components of canopy structural diversity (canopy height, cover and vertical complexity) using linear mixed-effects models. In these models, plot identity was included as a random effect to account for spatial dependence among quadrats nested in plots. To avoid multicollinearity in multivariate linear mixed-effects models, we excluded highly correlated variable combinations (*r* > 0.7, see Supplementary Table 5)^12^. Model selection was performed using the Akaike Information Criterion (AIC), retaining all models within ΔAIC ≤ 2 from the optimal model. The climatic variables featured in these retained models were identified as key determinants. Analyses were implemented with the ‘lme4’ package for model fitting^65^ and ‘MuMIn’ package^66^ for model selection.

Subsequently, we assessed bivariate relationships between productivity and two predictor groups—species richness and components of canopy structural diversity—across biomes using linear mixed-effects models, with plot identity as a random effect. We then analyzed within-plot relationships using generalized least squares models in the ‘nlme’ R package^67^, comparing models with and without spatial autocorrelation structures^68^ and selecting the best-fitting models based on AIC (Supplementary Data 1). For all models, we quantified explained variance by calculating marginal *R*^2^ (for fixed effects) and conditional *R*^2^ (for both fixed and random effects) using the ‘MuMIn’ R package^66^.

To assess the direct and indirect effects of climate, natural disturbance and species richness on forest productivity (Fig. 1), we employed piecewise structural equation models (SEMs) implemented in the ‘piecewiseSEM’ package^69^. In the SEM framework, plot identity (nested within biome identity where applicable) was treated as a random effect, and local topography and natural disturbance were included as fixed effects, given their potential to influence productivity both directly and indirectly—via effects on species diversity and components of canopy structural diversity (Fig. 1). To reduce the number of climatic variables and avoid multicollinearity, we performed a principal component analysis (PCA) and retained the first axis of the PCA (PC1) (70.3% variance explained; Supplementary Table 6) as an integrated representation of the full climate variable set. To represent the collective influence of topography, we also created a composite variable “Topography”. Key topographic variables were first identified through AIC-based model selection on multiple linear regressions with productivity. As convexity, slope, and topographic wetness index consistently improved model performance, the “Topography” composite variable was constructed as a weighted linear combination, using the standardized coefficients from the best AIC-selected regression model as weights^69^. Starting with a fully saturated model, non-significant pathways (*p* > 0.05) were sequentially eliminated until a parsimonious model with the lowest AIC was obtained. Likelihood ratio tests were employed to assess the significance of random effects (biome identity was not significant (Supplementary Table 7) and was excluded from the model), and overall model fit was evaluated using Fisher’s C statistic. Prior to analyses, we log-transformed productivity and logit-transformed canopy cover to mitigate heteroskedasticity, and standardized all variables to unit variance for direct effect size comparison in SEM models.

### Reporting summary

Further information on research design is available in the Nature Portfolio Reporting Summary linked to this article.

## Supplementary information

Supplementary Information

Description of Additional Supplementary Files

Supplementary Data 1

Supplementary Data 2

Reporting Summary

Transparent Peer Review file

## Source data

Source Data

## Data Availability

The data obtained from the generalized least squares models fitted to log-transformed above-ground net primary productivity are provided in Supplementary Data 1. For each model, the following information is included: slope, lower and upper confidence intervals (CIs), p-value, AIC, model type and the optimal model of predictors. The predictor assessed within each forest plot include species richness, mean outer canopy height, logit-transformed canopy cover, foliage height diversity. Site-specific and species-specific allometric equations for aboveground biomass estimation are provided in Supplementary Data 2. The quadrat-level forest productivity, species diversity, components of canopy structural diversity, topography and climate data generated in this study have been deposited in the Zenodo^70^ at: (10.5281/zenodo.18182641) and in Code Ocean at (10.24433/CO.5876339.v1). Source data are provided with this paper.

## Code availability

The programs used to generate all the results are made with R. Analysis R scripts are available at the Zenodo^70^ repository: (10.5281/zenodo.18182641) and Code Ocean repository: (10.24433/CO.5876339.v1).
