# Supplementary material for: Canopy structural diversity mediates the effect of climate on primary productivity in forests
Source: Nat Commun. 2026 Jul 11;17:8552. doi: 10.1038/s41467-026-75043-3 (PMC13484487; doi:10.1038/s41467-026-75043-3)
Supplement: Supplementary file 1 [file 41467_2026_75043_MOESM1_ESM.pdf]

Supplementary Information

Canopy structural diversity mediates the effect of climate on primary productivity in forests

Xiaoxia Yi[1,2,3], Grégoire Vincent[4], Tianyu Hu[1,2], Jianli Liu[5], James A. Lutz[6,7], Haibao Ren[1,2], Lei Chen[1,2], David F. R. P. Burslem[8], Joseph D. Birch[6,7], Min Cao[9,10], James W. Dalling[11,12], David A. Coomes[13], Géraldine Derroire[14,15,16], Hu Du[17], Shuai Fang[18,19], Guangze Jin[20], Jinbo Li[21], Fei Lin[18,19], Hongwei Ni[22], Michael J. O'Brien[23], Guochun Shen[24], Xihua Wang[24], Xingyu Wang[1,2], Jie Yang[9,10], Fuping Zeng[17], Daoguang Zhu[21],Yan Zhu[1,2], Yanjun Su[1,2], Qinghua Guo[25], Keping Ma[1,2,3], Xiangcheng Mi[1,2*]

1. Zhejiang Qianjiangyuan Forest Biodiversity National Observation and Research Station, Key Laboratory of Vegetation and Environmental Change, Institute of Botany, Chinese Academy of Sciences, Beijing 100093, China
2. China National Botanical Garden, Beijing 100093, China;
3. University of the Chinese Academy of Sciences, Beijing 100049, China
4. AMAP, Univ. Montpellier, CIRAD, CNRS, INRAE, IRD, Montpellier, France
5. College of Geography and Geomatics, Xuchang University, Xuchang 461000, China;
6. Wildland Resources Department, Utah State University, 5230 Old Main Hill, Logan, UT 84322-5230, USA.
7. The Ecology Center, Utah State University, 5230 Old Main Hill, Logan, UT 84322-5230, USA.
8. School of Biological Sciences, University of Aberdeen, Cruickshank Building, St

Machar Drive, Aberdeen AB24 3UU, UK
9. CAS Key Laboratory of Tropical Forest Ecology, Xishuangbanna Tropical Botanical Garden, Chinese Academy of Sciences, Kunming 650223, China
10. Center of Conservation Biology, Core Botanical Gardens, Chinese Academy of Sciences, Wuhan 430074, China
11. Department of Plant Biology, University of Illinois at Urbana-Champaign, Urbana, Illinois 61801, USA;
12. Smithsonian Tropical Research Institute, Apartado 0843–03092, Balboa, Panama
13. University of Cambridge, Cambridge, UK
14. Cirad, UMR EcoFoG (AgroParistech, CNRS, INRAE, Université des Antilles, Université de la Guyane), Campus Agronomique, Kourou, French Guiana
15. Cirad, UPR Forêts et Sociétés, University of Montpellier, Montpellier, France;
16. University of Brasilia, Department of Forestry, Campus Darcy Ribeiro, Brasília 70.900-910, Brazil
17. Key Laboratory of Agro-ecological Processes in Subtropical Region, Institute of Subtropical Agriculture, Chinese Academy of Sciences, Changsha, Hunan 410125, China
18. CAS Key Laboratory of Forest Ecology and Silviculture, Institute of Applied Ecology, Chinese Academy of Sciences, Shenyang, 110016, China
19. Key Laboratory of Terrestrial Ecosystem Carbon Neutrality, Liaoning Province, China
20. Center for Ecological Research, Northeast Forestry University, Harbin 150040,

China

21. Institute of Natural Resources and Ecology, Heilongjiang Academy of Sciences, Harbin 150040, China

22. Heilongjiang Academy of Forestry, Harbin 150081, China

23. Estación Experimental de Zonas Áridas, Consejo Superior de Investigaciones Científicas, Almería, Spain

24. Zhejiang Tiantong Forest Ecosystem National Observation and Research Station, School of Ecological and Environmental Sciences, East China Normal University, Shanghai 200241, China

25. Institute of Ecology, College of Urban and Environmental Science, Peking University, Beijing 100871, China

Correspondence author: Xiangcheng Mi: mixiangcheng@ibcas.ac.cn;

Table of Contents:

2········ Supplementary figure 1

3. ········Supplementary tables Table S1-S10

30········Supplementary Methods -*Evaluation of the impact of sensor characteristics on canopy structural metrics*

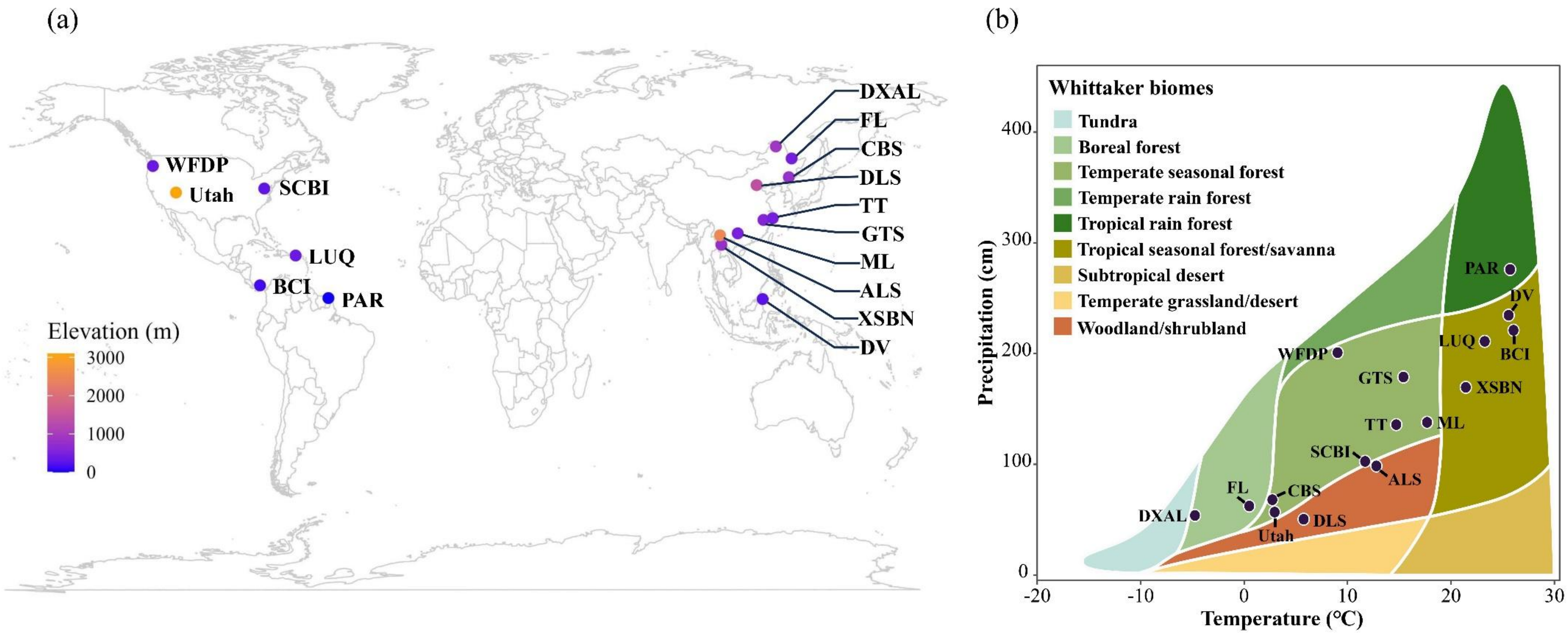

Supplementary Figure 1. A global map depicting the spatial distribution of the 16 forest dynamics plots in the study (a) **and Whittaker biome plot showing the mean annual temperature (°C) and mean annual precipitation (cm) of the plots** (b). Full plot names are listed in Supplementary Table 1. World base map is made with Natural Earth.

Supplementary Tables

Supplementary Table 1| Summary of forest plot characteristics and census intervals.

Plot name	Code	Latitude (°N)	Longitude (°E)	Plot size (ha)	Number of 50 m × 50 m quadrats	Number of 100 m × 100 m quadrats	Forest types	Census period
Danum valley	DV	5.102	117.688	50	200	50	Tropical rainforest	2011-2019
Paracou[1]	PAR	5.258	-52.937	25	100	25	Tropical rainforest	2015-2020[2]
Barro Colorado Island[3]	BCI	9.154	-79.846	50	200	50	Tropical moist deciduous forest	2010-2015[4, 5]
Luquillo[6]	LUQ	18.326	-65.816	16	60	15	Tropical rainforest	2011-2016
Xishuangbanna	XSBN	21.612	101.574	20	80	20	Tropical seasonal rainforest	2012-2017
Ailaoshan	ALS	24.536	101.028	20	80	20	Subtropical forest	2014-2019
Mulun	ML	25.133	108.012	25	100	25	Subtropical Karst forest	2014-2019
Gutianshan	GTS	29.250	118.117	24	96	24	Subtropical forest	2010-2015
Tiantong	TT	29.812	121.783	20	80	20	Subtropical forest	2010-2015
Utah[7, 8]	Utah	37.662	-112.853	15.32	27	6	Temperate forest	2014-2019
Smithsonian Conservation Biology	SCBI	38.894	-78.145	25.6	96	24	Temperate forest	2013-2018

Institute[9]								
Donglingshan	DLS	39.957	115.425	20	49	9	Temperate forest	2010-2015
Changbaishan	CBS	42.383	128.083	25	100	25	Temperate forest	2014-2019
Wind River[10, 11]	WFDP	45.820	-121.956	27.2	96	27.2	Temperate forest	2011-2016
Fenglin	FL	48.131	129.194	30	120	30	Temperate forest	2009-2019
Daxinganling	DXAL	51.817	122.998	25	84	18	Boreal forest	2014-2018

Supplementary Table 2. Results of linear mixed effect models used to predict forest productivity, species richness and canopy structural diversity based on combinations of climatic variables at the subplot scale (50 m × 50 m).

Forest productivity was quantified as above-ground net primary productivity, canopy height was measured using mean outer canopy height, and vertical structural complexity was assessed using foliage height diversity (See Methods). Key to abbreviations: MAP, mean annual precipitation; AI, aridity index; MAPcv, precipitation seasonality; MAT_grow, mean temperature in growing season; SR, solar radiation; R^2_M (Marginal R^2) is the proportion of variance explained by fixed effect variables and R^2_C (conditional R^2) is the proportion of variance explained by both fixed and random effect variables.

Response variables	Predictor variables	R^2_M	R^2_C	AIC	Δ AIC
Forest productivity	MAT_grow	0.38	0.54	3323.98	0.00
	MAT_grow + MAP	0.40	0.54	3325.63	1.65
	MAT_grow + MAPcv	0.42	0.55	3325.97	1.99
Species richness	MAT_grow	0.69	0.90	1020.07	0.00
Canopy height	MAT_grow	0.46	0.81	2064.49	0.00
	MAT_grow + AI	0.49	0.80	2065.26	0.77
	MAP	0.42	0.80	2065.27	0.77
	MAT_grow + MAP	0.48	0.81	2065.59	1.09
	MAT_grow + MAP + AI	0.47	0.80	2065.62	1.12
Canopy cover	MAT_grow + MAP + AI + SR	0.37	0.93	824.92	0.00
	MAT_grow	0.28	0.93	825.24	0.32
	MAP	0.22	0.93	826.33	1.41
Vertical structural complexity	MAP	0.35	0.88	1389.22	0.00
	AI	0.27	0.87	1390.44	1.22
	MAT_grow + MAP + AI	0.39	0.89	1390.82	1.60

	+ SR				
	MAP + AI	0.33	0.88	1391.02	1.80

Supplementary Table 3| Summary results of linear mixed-effects models for relationships between forest productivity and species richness, canopy height, canopy cover, vertical complexity, natural disturbance and climate at 50 m × 50 m scale. Statistical tests were two-sided, and no adjustments were made for multiple comparisons.

**Regressions**				
**Productivity ~ Predictor**	Standardized Estimate	SE	t	P
Species richness	0.254	0.047	5.382	<0.000
Canopy height	0.470	0.033	14.415	<0.000
Canopy cover	0.829	0.047	17.704	<0.000
Vertical complexity	0.385	0.042	9.261	<0.000
Natural disturbance	0.273	0.014	18.981	<0.000
Climate	0.683	0.118	5.794	<0.000

Supplementary Table 4. Flight specifications of the lidar data collection of the 16 forest plots (the code of each plot is consistent with Table S1).

Plot name	Lidar sensor	Date of survey	Wavelength (nm)	Beam divergence (mrad)	Flight height (m)	Pulse density (pulses/m²)	Scanning area/total area (ha)
Danum Valley	Leica ALS50-II	November 2014	905	0.22	2000	5.2	50/50
Paracou	Riegl LMS-Q780	October 2015	1064	0.25	800	28.8	25/25
Barro Colorado Island	Optech ALTM Gemini	August-September 2009	1064	0.25 × 0.8	457.2	13.2	50/50
Luquillo[12]	Riegl VQ-480i	March 2017	1550	0.3	335	25.0	16/16
Xishuangbanna	RIEGL VUX-1LR	October 2015	1550	0.5	350	20.5	20/20
Ailaoshan	Riegl VUX-1LR	October 2015	1550	0.5	350	46.2	20/20
Mulun	Sky-Lark	November 2019	1550	0.35	200	4.6	25/25
Gutianshan	Riegl VUX-1LR	September 2016	1550	0.5	200	11.2	24/24
Tiantong	RIEGL miniVUX-1UAV	April 2021	905	1.6 × 0.5	100	111.0	20/20
Utah	Optech Galaxy Prime	March 2018	1064	0.25	1450	8.0	15.32/15.32
Smithsonian Conservation Biology Institute[13]	Riegl LMS-Q780	July 2016	1064	0.25	1000	3.4	25.6/25.6
Donglingshan	Riegl VUX-1 LR	September 2018	1550	0.5	250	84.9	12.25/20

Changbaishan	Riegl VUX-1LR	August 2015	1550	0.5	100	54.4	25/25
Wind River	Leica ALS50 II	September 2011	1064	0.22	900	38.6	27.2/27.2
Fenglin	RIEGL miniVUX-1	July 2018	905	1.6 × 0.5	100	17.9	30/20
Daxinganling	LiAir 200 (Hesai Pandar40)	September 2019	905	3	120	72.6	21/25

Supplementary Table 5| Pearson's correlation coefficients of all climatic variables used in this study (see Supplementary Table 10 for full results). $r > 0.7$ are shown in bold. $^*p < 0.05$, $^{**}p < 0.01$, $^{***}p < 0.001$. Statistical tests were two-sided, and no adjustments were made for multiple comparisons.

	MAP	AI	MAPcv	MAT_grow
AI	**0.91***			
MAPcv	-0.49	-0.35		
MAT_grow	**0.79***	0.54*	-0.31	
SR	0.57*	0.21	-0.48	**0.72**

Key to abbreviations: MAP, mean annual precipitation; AI, aridity index; MAPcv, precipitation seasonality; MAT_grow, mean temperature in growing season; SR, solar radiation.

Supplementary Table 6| Loadings of the principal component analysis of six climatic variables in this study

Climatic variables	PC1	PC2
	(71.5%)	(15.9%)
Mean annual precipitation	0.52	0.17
Aridity index	0.42	0.56
Precipitation seasonality	-0.37	-0.37
Mean temperature in growing season	0.47	-0.44
Solar radiation	0.44	-0.57

The percentages below PC1 and PC2 indicates the proportion of the total variance in the climatic data that is accounted for by Principal Components 1 and 2.

Supplementary Table 7| Likelihood ratio test of piecewise structural equation models

Model	Random effects	AIC	BIC	Fisher C	Fisher C Difference	DF difference	P value
Model 1	plot	71.902	248.701	5.902			
Model 2	biome/plot	81.902	285.489	5.902	0	0	1

Models 1 and 2 share same set of fixed effects as the optimal model but differ in random effects. “biome/plot” indicates plot identity is nested within biome identity.

Supplementary Table 8| Summary results of linear mixed-effects models for the effects of individual climate variables on forest productivity, species richness and canopy structural diversity at 50 m × 50 m scale. Statistical tests were two-sided, and no adjustments were made for multiple comparisons.

**Regressions**				
Response ~ Predictor	Standardized Estimate	SE	t	P
**Productivity~**				
MAP	0.565	0.115	4.895	<0.000
AI	0.388	0.138	2.801	0.005
MAPcv	-0.335	0.163	-2.053	0.040
MAT_grow	0.624	0.106	5.912	<0.000
SR	0.513	0.147	3.497	<0.000
**Species richness~**				
MAP	0.712	0.160	4.447	<0.000
AI	0.499	0.183	2.727	0.006
MAPcv	-0.283	0.233	-1.217	0.224
MAT_grow	0.852	0.123	6.927	<0.000
SR	0.590	0.212	2.783	0.005
**Canopy height~**				
MAP	0.659	0.158	4.169	<0.000
AI	0.531	0.162	3.276	0.001
MAPcv	-0.169	0.228	-0.741	0.459
MAT_grow	0.699	0.160	4.356	<0.000
SR	0.410	0.226	1.810	0.070
**Canopy cover~**				
MAP	0.534	0.239	2.234	0.026
AI	0.429	0.226	1.900	0.057
MAPcv	0.005	0.274	0.018	0.986
MAT_grow	0.607	0.240	2.532	0.011
SR	0.410	0.275	1.491	0.136
**Vertical complexity~**				
MAP	0.613	0.191	3.219	0.001

AI	0.528	0.180	2.940	0.003
MAPcv	-0.223	0.240	-0.932	0.352
MAT_grow	0.565	0.213	2.647	0.008
SR	0.375	0.247	1.517	0.129

Key to abbreviations: MAP, mean annual precipitation; AI, aridity index; MAPcv, precipitation seasonality; MAT_grow, mean temperature in growing season; SR, solar radiation.

Supplementary Table 9| Piecewise structural equation model output for the effects of species richness, canopy structural diversity, natural disturbance, topography and climate on forest productivity at 50 m × 50 m scale. Statistical tests were two-sided, and no adjustments were made for multiple comparisons.

**Regressions**				
**Response ~ Predictor**	Standardized Estimate	SE	DF	P
**Productivity~**				
Climate	0.196	0.057	14	0.116
Natural disturbance	0.244	0.007	1546	0.000
Topography	0.138	0.103	1546	0.000
Species richness	0.126	0.001	1546	0.002
Canopy cover	0.175	0.009	1546	0.000
Canopy height	0.224	0.002	1546	0.000
Vertical complexity	0.234	0.029	1546	0.000
**Species richness~**				
Climate	0.780	2.955	14	0.000
**Canopy cover~**				
Climate	0.511	0.402	14	0.028
Natural disturbance	0.068	0.021	1549	0.000
Topography	0.084	0.336	1549	0.000
Species richness	0.156	0.003	1549	0.000
**Canopy height~**				
Climate	0.571	1.492	14	0.005
Natural disturbance	0.052	0.101	1550	0.000
Species richness	0.124	0.016	1550	0.000
**Vertical complexity~**				
Climate	0.406	0.133	14	0.060
Topography	-0.065	0.095	1550	0.000
Species richness	0.269	0.001	1550	0.000

**Variances explained by predictor**	Estimate	DF	P

**Canopy cover~~**			
Canopy height	0.522	1568	0.000
Vertical complexity	0.233	1568	0.000
**Canopy height~~**			
Vertical complexity	0.382	1568	0.000

Supplementary Table 10| Summary results of Pearson correlation coefficients among climatic variables. Statistical tests were two-sided, and no adjustments were made for multiple comparisons.

**Correlation coefficients**	Standardized Estimate	SE	t	P
**MAP~~**				
AI	0.911	0.110	8.279	<0.000
MAPcv	-0.489	0.233	-2.099	0.054
MAT_grow	0.794	0.163	4.881	<0.000
SR	0.569	0.220	2.588	0.021
**AI~~**				
MAPcv	-0.350	0.250	-1.400	0.183
MAT_grow	0.544	0.224	2.428	0.029
SR	0.205	0.262	0.785	0.446
**MAPcv~~**				
MAT_grow	-0.307	0.254	-1.205	0.248
SR	-0.479	0.235	-2.044	0.060
**MAT_grow~~**				
SR	0.721	0.185	3.892	0.002

Key to abbreviations: MAP, mean annual precipitation; AI, aridity index; MAPcv, precipitation seasonality; MAT_grow, mean temperature in growing season; SR, solar radiation.

Supplementary Methods

Evaluation of the impact of sensor characteristics on canopy structural diversity

To assess the robustness of LiDAR-derived canopy structural metrics to sensor characteristics, we leveraged multi-sensor data from a 2-ha tropical forest plot (Paracou, French Guiana) scanned with three distinct airborne LiDAR systems (Supplementary Table 11) in a recent study[23]. For each sensor, we calculated three components of structural diversity from first returns: mean outer canopy height, canopy cover and foliage height diversity (see Methods) at two spatial scales (10 m × 10 m and 25 m × 25 m). We quantified cross-sensor agreement using Pearson's correlation coefficients. Our results revealed moderate agreement at finer scales (10 m × 10 m: $0.56 < r < 0.85$; Supplementary Table 12) but strong consistency across all metrics at coarser scales (25 m× 25 m: $r > 0.92$; Supplementary Table 13). Results confirmed that LiDAR-derived structural metrics are robust to sensor-specific variations at scales ≥25 × 25 m, aligning with prior evidence that larger spatial scales mitigate sensor biases even under low point densities[24].

Supplementary Table 11| Technical specifications for the three lidar sensors and data of the 2-ha plot

Characteristic	LMSQ780	VUX-1UAV	MiniVux-1 UAV
Wavelength (nm)	1064 nm	1550 nm	905 nm
Beam divergence (mrad)	≤0.25	≤0.5	≤1.6×0.5
Flight height (m)	904 m	118 m	82 m
Footprint diameter (cm)	22.5 cm	5 cm	16×5 cm
Pulse density (pulses/m²)	34.5	144.4	219.3

Supplementary Table 12| Pearson's correlation coefficients of mean outer canopy height, canopy cover and foliage height diversity derived from three lidar sensors at a 10 m × 10 m scale.

Lidar metrics	Sensors	LMSQ780	VUX-1UAV	MiniVux-1UAV
Mean outer canopy height	VUX-1UAV	0.983		
	MiniVux-1UAV	0.986	0.992	1.000
Canopy cover	VUX-1UAV	0.887		
	MiniVux-1UAV	0.886	0.880	1.000
Foliage height diversity	VUX-1UAV	0.566		
	MiniVux-1UAV	0.591	0.850	1.000

Supplementary Table 13| Pearson's correlation coefficients of mean outer canopy height, canopy cover and foliage height diversity derived from three lidar sensors at the scale of 25 m × 25 m

Lidar metrics	Sensors	LMSQ780	VUX-1UAV	MiniVux-1UAV
Mean outer canopy height	VUX-1UAV	0.955		
	MiniVux-1UAV	0.965	0.992	1.000
Canopy cover	VUX-1UAV	0.964		
	MiniVux-1UAV	0.926	0.937	1.000
Foliage height diversity	VUX-1UAV	0.989		
	MiniVux-1UAV	0.999	0.996	1.000

References:

1. Gourlet-Fleury S., Guehl J.-M., Laroussinie O. E. *Ecology and management of a neotropical rainforest: lessons drawn from Paracou, a long-term experimental research site in French Guiana.* Elsevier (2004).

2. Guyafor. DataBase of the French Guyana Permanent Plot Network. Cirad-CNRS-ONF, http://www.ecofog.gf/spip.php?article364 (2023).

3. Hubbell S. P., *et al.* Light-Gap disturbances, recruitment limitation, and tree diversity in a neotropical forest. *Science* **283**, 554-557 (1999).

4. Condit R., Perez R., Aguilar S., Lao S., Foster R., Hubbell S. P. Complete data from the Barro Colorado 50-ha plot: 423617 trees, 35 years, (2019 version.) (2019).

5. Condit R., Perez R., Aguilar S., Lao S., Foster R., Hubbell S. P. BCI 50-ha plot taxonomy, (2019 version.) (2019).

6. Zimmerman J. K., Comita L. S., Thompson J., Uriarte M., Brokaw N. Patch dynamics and community metastability of a subtropical forest: compound effects of natural disturbance and human land use. *Landsc. Ecol.* **25**, 1099-1111 (2010).

7. Furniss T. J., Larson A. J., Lutz J. A. Reconciling niches and neutrality in a subalpine temperate forest. *Ecosphere* **8**, e01847 (2017).

8. Lutz J. A. The evolution of long-term data for forestry: large temperate research plots in an era of global change. *Northwest Sci.* **89**, 255-269 (2015).

9. Bourg N. A., McShea W. J., Thompson J. R., McGarvey J. C., Shen X. Initial census, woody seedling, seed rain, and stand structure data for the SCBI SIGEO Large Forest Dynamics Plot. *Ecology* **94**, 2111-2112 (2013).

10. Lutz J. A., Larson A. J., Freund J. A., Swanson M. E., Bible K. J. The importance of large-diameter trees to forest structural heterogeneity. *PLoS One* **8**, e82784 (2013).

11. Lutz J. A., *et al.* Spatially nonrandom tree mortality and ingrowth maintain equilibrium pattern in an old-growth Pseudotsuga–Tsuga forest. *Ecology* **95**, 2047-2054 (2014).

12. Cook B. D., *et al.* NASA Goddard's LiDAR, hyperspectral and thermal (G-LiHT) airborne imager. *Remote Sens.* **5**, 4045-4066 (2013).

13. National Ecological Observatory Network. Discrete return LiDAR point cloud

(DP1.30003.001).). https://doi.org/10.48443/6e8k-3343 (2021).

14. Xu Y., Zhang Y., Shi M., Lu Z. Environmental variation, functional diversity and identity predicting community biomass in an old-growth subtropical broad-leaved forest. *Glob. Ecol. Conserv.* **23**, e01093 (2020).

15. Wang Z. C.*, et al.* Allometric models of major tree species and forest biomass in Guangxi. *Acta Ecol. Sin.* **35**, 4462-4472 (2015).

16. Lin D., Lai J., Muller-Landau H. C., Mi X., Ma K. Topographic variation in aboveground biomass in a subtropical evergreen broad-Leaved forest in China. *PLoS One* **7**, e48244 (2012).

17. Ren S.*, et al.* The driving factors of subtropical mature forest productivity: stand structure matters. *Forests* **12**, 998 (2021).

18. Wang S., Qi G., Knapp B. O. Topography affects tree species distribution and biomass variation in a warm temperate, secondary forest. *Forests* **10**, 895 (2019).

19. Yuan Z.*, et al.* Multiple metrics of diversity have different effects on temperate forest functioning over succession. *Oecologia* **182**, 1175-1185 (2016).

20. Chen C. G., Zhu J. F. *A Handbook for Main Tree Species Biomass in Northeast China*. China Forestry Press (1989).

21. Li X. N., Guo Q. X., Wang X. Allometry of understory tree species in a natural secondary forest in northeast China. *Sci. Silvae Sin.* **46**, 22-32 (2010).

22. Fu Y. Y. Remote sensing estimation and simulation of major tree species aboveground biomass and density in the forest region of northeast China. Northeast Normal University, China (2020).

23. Vincent G.*, et al.* Multi-sensor airborne lidar requires intercalibration for consistent estimation of light attenuation and plant area density. *Remote Sens. Environ.* **286**, 113442 (2023).

24. Kamoske A. G., Dahlin K. M., Stark S. C., Serbin S. P. Leaf area density from airborne LiDAR: Comparing sensors and resolutions in a temperate broadleaf forest ecosystem. *For. Ecol. Manage.* **433**, 364-375 (2019).
