# Supplementary material for: Canopy structural diversity mediates the effect of climate on primary productivity in forests
Source: Nat Commun. 2026 Jul 11;17:8552. doi: 10.1038/s41467-026-75043-3 (PMC13484487; doi:10.1038/s41467-026-75043-3)
Supplement: Supplementary file 2 [file 41467_2026_75043_MOESM2_ESM.pdf]

# Description of Additional Supplementary Files

**File: Supplementary Data 1**

Description: Output from the fits of the generalized least squares models of log-transformed above-ground net primary productivity as a function of species richness, mean outer canopy height, logit-transformed canopy cover, foliage height diversity within each forest plot. Note: Non-spatial and spatial models denote generalized least-squares models that either exclude or incorporate an autocorrelation structure to account for spatial autocorrelation among quadrats (See details in 'Materials and methods' section). CI-low and CI-high show the lower and upper confidence limits of slope coefficients. Significance levels: ns, nonsignificant at $p \geq 0.10$, †$p < 0.10$, *$p < 0.05$, **$p < 0.01$, ***$p < 0.001$. The model with the lowest AIC score was selected as the optimal model. Statistical tests were two-sided, and no adjustments were made for multiple comparisons.

**File Name: Supplementary Data 2**

Description: Site-specific and species-specific allometric equations for aboveground biomass estimation. AGB: aboveground dry biomass (kg), D: diameter at breast height (cm), H: tree height (m).
