# Supplementary material for: Canopy structural diversity mediates the effect of climate on primary productivity in forests
Source: Nat Commun. 2026 Jul 11;17:8552. doi: 10.1038/s41467-026-75043-3 (PMC13484487; doi:10.1038/s41467-026-75043-3)
Supplement: Supplementary file 3 [file 41467_2026_75043_MOESM5_ESM.pdf]

nature portfolio

Corresponding author(s): Xiangcheng Mi

Last updated by author(s): Mar 26, 2026

Reporting Summary

Nature Portfolio wishes to improve the reproducibility of the work that we publish. This form provides structure for consistency and transparency in reporting. For further information on Nature Portfolio policies, see our Editorial Policies and the Editorial Policy Checklist.

Statistics

For all statistical analyses, confirm that the following items are present in the figure legend, table legend, main text, or Methods section.

n/a	Confirmed	
☐	☒	The exact sample size (*n*) for each experimental group/condition, given as a discrete number and unit of measurement
☐	☒	A statement on whether measurements were taken from distinct samples or whether the same sample was measured repeatedly
☐	☒	The statistical test(s) used AND whether they are one- or two-sided<br>*Only common tests should be described solely by name; describe more complex techniques in the Methods section.*
☐	☒	A description of all covariates tested
☐	☒	A description of any assumptions or corrections, such as tests of normality and adjustment for multiple comparisons
☐	☒	A full description of the statistical parameters including central tendency (e.g. means) or other basic estimates (e.g. regression coefficient) AND variation (e.g. standard deviation) or associated estimates of uncertainty (e.g. confidence intervals)
☐	☒	For null hypothesis testing, the test statistic (e.g. F, t, r) with confidence intervals, effect sizes, degrees of freedom and P value noted<br>*Give P values as exact values whenever suitable.*
☒	☐	For Bayesian analysis, information on the choice of priors and Markov chain Monte Carlo settings
☐	☒	For hierarchical and complex designs, identification of the appropriate level for tests and full reporting of outcomes
☐	☒	Estimates of effect sizes (e.g. Cohen's *d*, Pearson's *r*), indicating how they were calculated

Our web collection on statistics for biologists contains articles on many of the points above.

Software and code

Policy information about availability of computer code

Data collection	No software was used.
Data analysis	All analyses were done using the software environment R version 4.3.1. LiDAR metrics were calculated using the 'lidR' package 4.1.2. The aboveground biomass for temperate forest plots was estimated using the 'allodb' package v.0.0.1.9000 for North American plots, while aboveground biomass for tropical forest plots was calculated using the 'BIOMASS' package v. 2.1.11. Observed β-diversity values were calculated and standardized using 'vegan' R package v.2.6-4. Topographic variables were calculated using the 'RSAGA' package v.1.0.0 and the SAGA GIS software v. 2.1.4. Statistical analyses were done using 'nlme' package v.3.1-164, 'MuMln' package v.1.47.5 and 'piecewiseSEM' package 2.1.0. The R code for all analyses in this study is available at the Zenodo repository: https://doi.org/10.5281/zenodo.18182641 and Code Ocean repository: https://doi.org/10.24433/CO.5876339.v1.

For manuscripts utilizing custom algorithms or software that are central to the research but not yet described in published literature, software must be made available to editors and reviewers. We strongly encourage code deposition in a community repository (e.g. GitHub). See the Nature Portfolio guidelines for submitting code & software for further information.

Data

Policy information about availability of data

All manuscripts must include a data availability statement. This statement should provide the following information, where applicable:

- Accession codes, unique identifiers, or web links for publicly available datasets
- A description of any restrictions on data availability
- For clinical datasets or third party data, please ensure that the statement adheres to our policy

The data obtained from the generalized least squares models fitted to log-transformed above-ground net primary productivity are provided in Supplementary Data 1. For each model, the following information is included: slope, lower and upper confidence intervals (CIs), p-value, AIC, model type and the optimal model of predictors. The predictor assessed within each forest plot include species richness, mean outer canopy height, logit-transformed canopy cover, foliage height diversity. Site-specific and species-specific allometric equations for aboveground biomass estimation are provided in Supplementary Data 2. Raw data underlying all figures are available in the Source Data files accompanying this paper. The quadrat-level forest productivity, species diversity, components of canopy structural diversity, topography and climate data generated in this study have been deposited in the Zenodo at: https://doi.org/10.5281/zenodo.18182641, and in Code Ocean at https://doi.org/10.24433/CO.5876339.v1.

Research involving human participants, their data, or biological material

Policy information about studies with human participants or human data. See also policy information about sex, gender (identity/presentation), and sexual orientation and race, ethnicity and racism.

Reporting on sex and gender	NA
Reporting on race, ethnicity, or other socially relevant groupings	NA
Population characteristics	NA
Recruitment	NA
Ethics oversight	NA

Note that full information on the approval of the study protocol must also be provided in the manuscript.

Field-specific reporting

Please select the one below that is the best fit for your research. If you are not sure, read the appropriate sections before making your selection.

☐ Life sciences ☐ Behavioural & social sciences ☒ Ecological, evolutionary & environmental sciences

For a reference copy of the document with all sections, see nature.com/documents/nr-reporting-summary-flat.pdf

Ecological, evolutionary & environmental sciences study design

All studies must disclose on these points even when the disclosure is negative.

Study description	We quantified three core components of canopy structural diversity—including canopy cover, canopy height, and vertical complexity—across boreal, temperate, subtropical, and tropical natural forest biomes. Our study aimed to unravel (i) the indirect effects of climate on forest productivity mediated by shifts in canopy structural diversity and (ii) latitudinal gradients in productivity linked to the structural diversity. The results revealed strong correlations between forest productivity and all structural diversity components. Furthermore, these structural metrics exhibited strong associations with climatic variables. In total, we sampled 1568 50 m ×50 m non-overlapping quadrats at 16 natural forest plots (n = 1568) across five biomes, with 204 quadrats of two plots in boreal forests (n=204), 595 quadrats of seven in temperate seasonal forests (n=595), 129 quadrats of two plots in woodland/shrubland (n=129), 540 quadrats of four plots of tropical seasonal forest/savanna (n=540), and 100 quadrats of one plot in tropical rainforest (n=100). The number of replicates for each biome is the total number of forest quadrats. However, spatial autocorrelation among quadrats within individual plots may reduce the effective sample size below the nominal replicate count. To address this issue, within-plot analyses utilized generalized least squares (GLS) models to explicitly model spatial autocorrelation, while across-plot analyses employed piecewise structural equation models (SEMs). In the SEM framework, plot identity or plot identity nested with biome identity were included as random effects to explicitly control for spatial dependence among quadrats within individual plots.
Research sample	The research sample represents natural forests with minimal anthropogenic disturbance from the boreal, temperate, subtropical and tropical forests. The rationale for the sample choice was to sample forests that (1) do not show signs of anthropogenic disturbance that modify forest structure and hence mask climatic and topographic effects on forest structural diversity, (2) to cover a latitudinal gradients in climate and diversity of forest biomes and to (3) sample forests that represent dominant forest types in their respective biome. (4) had the two most recent censuses closest to the LiDAR survey dates in order to temporally align forest census data with

	the LiDAR data. Since study sites were distributed in Asia and North and South America, the number of sites was constrained by available resources in terms of time and available funds.
Sampling strategy	The 16 forest plots were selected to cover latitudinal gradients in climate and diversity of forest biomes. To get robust estimates of forest structure for each study plot, a systematic sampling grid with was laid out across the area to be investigated (sampling method here is systematic sampling). All 50 m ×50 m non-overlapping quadrats in each study plot were selected.
Data collection	At each forest plot, a systematic sampling grid was laid out across the plot to be investigated. The coordinates of four plot corners was transferred to a GPS receiver that was used to find the respective plot location in the forest. At each forest plot, airborne LiDAR sensors (see details in supplementary Table 4) were employed to measure the forest structure three-dimensionally. Ground-based forest censuses were conducted following a standardized protocol at 5-year intervals. In each census, all woody stems with a diameter at breast height (DBH) ≥ 1 cm were tagged, spatially mapped and identified to species. The Paracou plot constituted an exception, censusing only stems ≥ 10 cm DBH.
Timing and spatial scale	LiDAR data were collected between August 2009 - April 2021. Measurements at each site were made during the growing season in fully leaved conditions to enable comparability between evergreen and deciduous forest types. The spatial scale from which the data was taken is across-continents. Forest censuses were also conducted during the growing season between 2011-2020.
Data exclusions	No data was excluded from the analysis.
Reproducibility	This study is an observational study (not an experiment) based on a sampling approach. Coordinates of forest plot locations are given in the supplementary material (Supplementary Table 1) so that the study is reproducible. Laser scanner settings are described in detail in the supplementary material (Supplementary Table 4). The R code of all analyses in this study is available at the Zenodo repository: https://doi.org/10.5281/zenodo.18182641 and Code Ocean repository: https://doi.org/10.24433/CO.5876339.v1. All re-analyses proved the robustness of model outcomes and the resulting conclusions.
Randomization	Samples (50 m × 50 m forest quadrats) were allocated to forest plot and biome based on a published classification of biomes by Whittaker (1962).
Blinding	To avoid bias during data acquisition, all 50 m ×50 m quadrats within each plot were scanned, while all stems with with a diameter at breast height (DBH) ≥ 1 cm were censused. Blinding during data analysis was not relevant, because the data was processed using an automated algorithm (see code availability statement).

Did the study involve field work? ☒ Yes ☐ No

Field work, collection and transport

Field conditions	Field work was carried out with wind speeds below 10 m per second to avoid measurement errors due to moving tree canopies. Field work was not carried out during rain, because the measurement device is not waterproof and because raindrops might cause stray points in the resulting point clouds. Temperature and humidity varied depending on the climate characteristics of the study sites.
Location	The study was conducted at 16 different study sites spread across continents. Information on site location (longitude and latitude), plot size, forest type and census period is given in Supplementary Table 1 of the manuscript.
Access & import/export	The collected data was stored in electronical format on SD Cards. No physical samples were collected and imported or exported. Research permits were granted by Qianjiangyuan National Park, the Utah Agricultural Experiment Station, the Gifford Pinchot National Forest and the U.S. Forest Service Wind River Field Station.
Disturbance	The study did not cause any disturbances, because no physical samples were collected. The use of a air laser scanner is entirely undisturbing.

Reporting for specific materials, systems and methods

We require information from authors about some types of materials, experimental systems and methods used in many studies. Here, indicate whether each material, system or method listed is relevant to your study. If you are not sure if a list item applies to your research, read the appropriate section before selecting a response.

Materials & experimental systems

n/a	Involved in the study
☒	☐ Antibodies
☒	☐ Eukaryotic cell lines
☒	☐ Palaeontology and archaeology
☒	☐ Animals and other organisms
☒	☐ Clinical data
☒	☐ Dual use research of concern
☒	☐ Plants

Methods

n/a	Involved in the study
☒	☐ ChIP-seq
☒	☐ Flow cytometry
☒	☐ MRI-based neuroimaging

Plants

Seed stocks	NA
Novel plant genotypes	NA
Authentication	NA
