# Supplementary material for: Canopy structural diversity mediates the effect of climate on primary productivity in forests
Source: Nat Commun. 2026 Jul 11;17:8552. doi: 10.1038/s41467-026-75043-3 (PMC13484487; doi:10.1038/s41467-026-75043-3)
Supplement: Supplementary file 4 [file 41467_2026_75043_MOESM6_ESM.pdf]

nature portfolio

Peer Review File

Canopy structural diversity mediates the effect of climate on primary productivity in forests

Corresponding Author: Dr Xiangcheng Mi

This file contains all reviewer reports in order by version, followed by all author rebuttals in order by version.

Version 0:

Reviewer comments:

Reviewer #1

(Remarks to the Author)
This paper reports a very impressive dataset of coupled LiDAR and forest inventory data from two networks of large forest plots spanning latitudinal transects through Asia and the Americas. This dataset is coupled with climate variables derived from gridded products to examine drivers of spatial variation in productivity and, in particular, to investigate the role of canopy structure in affecting productivity.

Improving our capacity to understand the ecological significance of remotely sensed metrics of forest ecosystems is very important and timely in the face of global change and the need for large-scale ecological monitoring. This paper presents a dataset with substantial potential to generate critical insights. I very much appreciated the mammoth scope of the project and objectives of this study.

I have a few general concerns or queries about the manuscript along with a series of minor comments or suggestions, which I outline below. I hope the authors will find these comments helpful in revising their manuscript.

This study seeks to account for major drivers of forest productivity across a very large area, and considers climate, species richness, topography and canopy structure. One question that deserves some careful thought is whether there are other key drivers of productivity that need to be accounted for. Two main thoughts come to mind: soil fertility, and disturbance. Variation in soil fertility among plots (or within plots) does not seem to have been considered; could the authors please consider and comment on this omission?

On disturbance and accounting for forest age or developmental stage, it is clear that the forests have not been subject to strong recent anthropogenic disturbance (line 212). However, it would be helpful to consider the extent to which natural disturbances, such as fires and hurricanes, have affected the forests. For instance, I understand that the Luquillo plot, at least, has experienced substantial hurricane disturbance. I would expect that disturbance events (or regimes) and the resulting age or developmental stage of the stands might be expected to strongly influence both forest structure and productivity. If possible, it would be helpful to account for this both in the conceptual reasoning as well as formally in the statistical models.

I found some of the conceptual framing and conclusions did not appear to be fully supported by study. For example, the study is framed in terms of climate change. This choice seemed slightly strange to me. While I agree that the insights into forest ecosystem change under climate change are critical, the study does not investigate climate change and instead looks at spatial variation over broad climatic/latitudinal gradients. This line of interpretation is followed through to the conclusions of the paper, with a major finding reported to be that dynamics of forest structure will be capture climate change effects on productivity. From the results and reasoning provided, I was not entirely convinced that the observed spatial relationships with climate will necessarily reflect changes over time at a given location. Given the potential consequences of these conclusions (i.e., that these findings might inform policy and direct the allocation of monitoring efforts), I believe it is very important that these findings are robust and well-substantiated.

Similarly, in both the abstract and final paragraph in the discussion, there is a statement to the effect that the results emphasise ‘the importance of incorporating canopy structural diversity into conservation priority frameworks’. I found this conclusion somewhat unconvincing or open to misinterpretation. How do these results connect directly with conservation

objectives? I believe these statements ought to be tempered or the connections much more clearly explained.

A broader issue on framing: Canopy height, canopy cover and vertical structural complexity are presented in the introduction (lines 24-25) as measures of structural diversity. This is an issue I have seen elsewhere in the literature as well. I would argue that the latter captures a dimension of heterogeneity, but the first two might be more accurately described simply as measures of forest structure.

On one level, the writing is quite smooth. However, on closer reading, I found some of the writing and logic unclear. In general, precision and specificity could be improved. There are instances where unnecessary words are added that increase complexity and potentially cause confusion without aiding meaning. There is also some inconsistency in the terms given to metrics. Overall, these issues obscure the potential impact of the manuscript. I have pointed out some examples in my specific comments (below) and would recommend carefully revising the entire manuscript.

Minor comments

Line 9: In this context, the intended meaning of 'inter-calibrated' was unclear to me. Is this word necessary? If so, could it perhaps be further defined in the main text?

Line 11: I presume beyond their direct effects on 'forest productivity'.

Line 21-22: I think I follow the intended meaning, but this phrasing seems somewhat imprecise to me.

Line 34: This sentence is unclear (architectural mediator of what?).

Line 45: While plausible, this statement appears unsubstantiated. Is this intended as a hypothesis or as a substantiated statement?

Line 46: In what sense are these mechanisms synergistic?

Line 47: Partitioning of what?

Line 72: Two quadrat sizes are mentioned here and again in the methods section. However, it was not clear to me why two quadrat sizes were chosen and, indeed, how the two quadrat sizes were used. All analyses appear to use the 50 m x 50 m quadrats.

Line 129-131: This statement was unclear to me. Canopy height and cover are modelled in many DGVMs, for instance. It would help to be more specific about the kinds of ecosystem models and the kinds of metrics that are being referred to here.

Line 151-153: As for the previous comment, some clarification or greater specificity about the additional processes being proposed would be helpful. It is my understanding that models already exist that include some of these processes by which environmental conditions limit, for instance, the height and density of forest canopies.

Line 176: What is structural complementarity?

Line 197-199: I am not sure about this conclusion. This study examines change over space and not change over time. It is not clear to me that these patterns would necessarily be substitutable.

Methods

Line 217: I appreciate these tables, but it takes some effort to compare the timing of the forest inventories with the LiDAR measurements. I would recommend keeping the tables. However, would it be possible to also add a summary to the main text of how closely the two datasets are matched in time? It appears that most (but not all) overlap in time, with some LiDAR measurements occurring toward the beginning and others toward the end of the census interval.

Line 221: The phrasing here was slightly unclear me: smaller than what? I think this is simply referring to small plots.

Line 223: Would it be possible to clarify how many quadrats were at each plot? Perhaps a range could be added to the main text and this information also added to a new column in Supplementary Table 1.

In general, it would also help to clearly explain the spatial scale of analyses (i.e., plot or quadrat or both) and why this spatial scale was chosen or appropriate for the given analysis.

Line 240: Would it be possible to add some more details here: how closely is "closely aligned"?

Line 244-248: It would help to explain the reasoning behind this analysis; as it stands, it is not clear (e.g. what sampling bias exactly?). Also, more broadly, the rationale for the beta-diversity analysis was not well introduced.

Line 249: Was the entire area of each plot surveyed? This is currently not clear.

Line 264: Here and among the figures, there appears some inconsistency in the terminology being used for different metrics (e.g., vertical structural complexity, foliage height diversity; canopy height, mean outer canopy height). It is unclear if these are the same or different metrics, and it would help for this to be presented very clearly and consistently throughout.

Line 296: Could the meaning of 'altitude above channel' be clarified?

Line 314: Here I was slightly confused about the model selection. This seems to imply that multiple models were retained, and it is unclear how they were subsequently used.

Line 335: Please explain why and how the topographic variables were combined into a composite variable.

Line 340: Please explain why these transformations were appropriate.

Fig. 3: Please define the abbreviation CWP

Fig. 4: It seems common to emphasise whether relationships are positive or negative in structural equation models. However, that information is not emphasised here, and I wondered about that decision.

Supplementary Table 4: I see there are two LiDAR surveys of Barro Colorado Island. Perhaps I missed it, but it was unclear to me if and how the two surveys were used (i.e., were both surveys used and treated as separate datapoints, or was one chosen, or were the two surveys somehow combined?). Could this please be clarified?

(Remarks on code availability)

Reviewer #2

(Remarks to the Author)
This is a unique study that reassesses and rethinks the impact of climate change on forest productivity. In contrast to conventional approaches. The authors examine not only the direct effects of factors such as mean annual precipitation and mean annual temperature on biomass growth, but also the indirect effects of these factors through related forest characteristics such as species diversity, mean canopy height, canopy cover or leaf height diversity.
Using an exceptional dataset of plots from boreal to tropical forests, including savannas and scrubland, the authors are able to confirm their hypothesis that climate, species diversity and canopy structural diversity together drive the latitudinal gradient in forest productivity.
The data include inventory and airborne laser scanning data collected at 16 study sites between 2005 and 2020. Although the period of data collection differs between study sites, the authors have taken care to ensure comparability of the data, for example by ensuring that all data were collected during the growing season.
The data were analysed using a number of well-chosen statistical models, including linear mixed-effects models, generalised least squares models or stepwise structural equation models. The results clearly show that the indirect effects of climate on canopy structure diversity are significant in shaping forest productivity. The results support the conclusion that this effect should be taken into account when assessing forest responses to climate change, e.g. by including it in global vegetation models.
Overall, the manuscript is very well written and clear. I have only a few comments and suggestions:

1. Although the dataset is impressive and a result of the available data, it is not very clear to what extent the differences in the number of plots between biomes affect the results. Can you comment on potential biases?
2. Line 72: Why do you use different quadrat sizes (50 m x 50 m vs. 100 m x 100 m)?
3. Line 77: It seems that the allometric relations used are always valid for a given species. Do they not change for different sites?
4. Lines 104 - 106: while it is reasonable to focus on R marginal in the results to explain the fixed effects, R conditional is also presented in Figure 3. It might be good to refer to it and its meaning (fixed + random effect) in at least one sentence.
5. Line 122: The results suggest that the direct effects are not significant. Although I'm aware that the common use of statistical significance is problematic, I wonder if the quantitative difference between direct and indirect effects isn't worth discussing further.
6. L.160-164: I agree with the statement about the potential error in the magnitude of indirect pathways, but the statement that the qualitative conclusion is still valid sounds a bit sloppy. Is there anything that can be used to support this statement?
7. L. 189: Asymmetric competition between shade-tolerant understory and light-demanding canopy species? Maybe I am not getting your point here, but I would expect that competition for light would become less asymmetric and more symmetric in a situation where more light passes through the canopy and reaches the understorey.
8. L. 202: Forest resilience? Why?
9. L. 234: Does the number of unidentified taxa differ between biomes, and if so, does this affect the results?
10. Fig. 3: The use of the PCA scores of the plots as a proxy for the climate variable is not very clear (to me). Does this ordinal variable meet the requirements for using this linear model?
11. I know that a piecewise SEM requires more data than a normal SEM, but I do not know how many data are needed. Can you give some information?

(Remarks on code availability)

Line 14 produces a small error because data1 contains only 20 columns instead of 32.

It would be nice to add some code to show that you have tested the conditions of your statistical models, e.g. normality of residuals for GLM or a Shipley's test of directed separation for SEM (if that makes sense).

Reviewer #3

(Remarks to the Author)
The authors combined forest inventory data used to estimate biomass (ground truth) with Lidar derived canopy attributes (canopy height, cover and vertical heterogeneity) and worldclim2 database derived climate variables to model productivity changes as a function of plot-level species richness, species diversity (beta diversity), canopy attributes and climate. Authors reported a stronger relation between canopy structural attributes and productivity compared to direct climate-productivity relationship. I find the work interesting but I wonder the novelty in this work. The relationship between stand attributes and productivity has been well published. I understand that authors are taking a larger scale (Biome) approach but I'm not sure there is much new insights from this study. Below are a few additional comments;
1) The data involved in this study occur at different spatial scales; biomass was computed at plot-level (13 to 50 ha plots), the Lidar data has a resolution of 50m x 50m, the climate data had a resolution of 1 km2. Authors did not explain how they dealt with the differences in scale. Were the inventory plots spaced out 1 km apart to match the climate data?
2) Authors did not mention how old these forest stands are at the census time (2010-2019). This is important to understand how relevant the 1971-2000 climate data/reference is regarding biomass accumulation.
3) Authors mentioned that there were 16-forest dynamics plots but the Figure 2 data has several data points. There were also random mentions of quadrat with no explanation how this is connected with plots and the Figure 4 mentioned n=1568 quadrats.
4) I was also wondering why authors did not included historical disturbances in their model.
5) In the SEM (Figure 4a), which climate variable is represented? authors mentioned that they performed PCA and retained the first principal axis (supplement Table 7) - was any particular climate variable used in the SEM based on the PCA results?
6) Line 90; reference to supplementary Table 1 here is incorrect.

(Remarks on code availability)

Version 1:

Reviewer comments:

Reviewer #1

(Remarks to the Author)
I wish to thank the authors for their revisions in response to the previous round of reviews. I believe these revisions have improved the paper. Overall, I remain impressed by the LiDAR and associated forest inventory data, and I find the broad topic of the paper interesting.

However, I found the revised paper remained hard going in places. While the results are quite interesting, I found myself wondering about the direction of causation and whether the observed relationships between canopy structure and productivity are not surprising and would be expected to emerge by definition. This issue aside, my main concerns centre around the conceptual framing, in particular the way structural diversity is presented as a ‘mechanism’. As it is currently presented in the paper, I did not find this convincing and I believe that some further conceptual development is needed. What are the processes by which canopy structure ought to affect productivity (is this about space filling, partitioning of light environments within the canopy, something else, or all of the above?)? What is the evidence that canopy structure is a driver rather than passenger of productivity? The structural equation model is fine, but I think a deeper process-based explanation is needed; an explanation that draws upon resource partitioning or physiological trade-offs or other processes.

Related, a key message in the paper is that canopy structure needs to be considered in ecosystem models. I do not necessarily disagree. But the reasons *why* this complexity is needed are not clearly explained. To justify adding this kind of complexity in models, there ought to be a clear process-based pathway by which canopy structure represents a dynamic with meaningful consequences – alongside evidence of the consequence of not representing this dynamic within models. To reiterate, I think there probably is justification, I just do not think this is currently clearly articulated.

Some specific comments:

Introduction: I found the reasoning somewhat unclear or lacking precision in places. For example, on line 28, ‘physiological constraints’ is broad and somewhat unclear; could the authors explain what they mean with a little more precision? Similarly, on line 45, the ‘synergy between climate effects’ and what? And, on line 49, what is meant by ‘leaf types’? Is this referring to species that have different photosynthetic responses to light?

Lines 29-30: This implies that canopy structure generates light use efficiency. It would be helpful to more clearly spell out the role of physiological differences among trees.

Line 83-85: This biological/ecological interpretation of beta-diversity is not clear to me. This suggests that beta-diversity will, in itself, give insight into the dominance of fast-growing species. I do not see how this is possible. Could this be revised and clarified?

Line 96-97: Why test both aridity index (P/PET) and water balance (P - PET)? Unsurprisingly they are very tightly correlated (Supplementary Table 6) and have a similar interpretation. Unless there is a clear reason that I am missing, I would be inclined to choose and show one (probably the aridity index).

Line 124: Would this be expected to emerge by definition (i.e., given the way that disturbance has been characterised)?

Line 279: As flagged above, I do not follow this interpretation of beta-diversity (beta-diversity is a metric of species turnover but does not reveal the identity or functional characteristics of species).

Line 252: Am I correct in understanding that trees reaching the threshold size to be included in the census for the first time had their entire biomass considered as new growth? This would seem to overestimate biomass growth.

I may have missed it, but I could not see the data availability statement. Will the LiDAR data be made available?

(Remarks on code availability)

Reviewer #2

(Remarks to the Author)
Dear authors, thank you very much for carefully revising your manuscript. I appreciate the way you have addressed the comments and the effort you have made to clarify unclear sentences or content. I confirm that you have addressed all my comments in sufficient detail.

(Remarks on code availability)
I have also reviewed the R code you provided via Code Ocean. It now works flawlessly and is very helpful for understanding the methodology. I have no further comments.

Reviewer #3

(Remarks to the Author)
Authors addressed my earlier comments quite satisfactorily. I do think the subject is not excessively novel (see these two papers below), however, authors argue that dynamic global vegetation models (DGVMs) represent forest canopies with large-scale mean metrics like leaf area index and height so this work can change that by emphasizing the need to incorporate canopy structural diversity - is a good response.

Paquette, A. and Messier, C., 2011. The effect of biodiversity on tree productivity: from temperate to boreal forests. Global Ecology and Biogeography, 20(1), pp.170-180.

Anyomi, K.A., Raulier, F., Bergeron, Y. and Mailly, D., 2013. The predominance of stand composition and structure over direct climatic and site effects in explaining aspen (Populus tremuloides Michaux) site index within boreal and temperate forests of western Quebec, Canada. Forest ecology and management, 302, pp.390-403.

(Remarks on code availability)

Version 2:

Reviewer comments:

Reviewer #1

(Remarks to the Author)
I wish to thank the authors for carefully considering my comments and revising their manuscript accordingly. These revisions have satisfactorily addressed my concerns.

(Remarks on code availability)

Open Access This Peer Review File is licensed under a Creative Commons Attribution 4.0 International License, which permits use, sharing, adaptation, distribution and reproduction in any medium or format, as long as you give appropriate credit to the original author(s) and the source, provide a link to the Creative Commons license, and indicate if changes were made.
In cases where reviewers are anonymous, credit should be given to 'Anonymous Referee' and the source.
The images or other third party material in this Peer Review File are included in the article's Creative Commons license, unless indicated otherwise in a credit line to the material. If material is not included in the article's Creative Commons license and your intended use is not permitted by statutory regulation or exceeds the permitted use, you will need to obtain permission directly from the copyright holder.
To view a copy of this license, visit https://creativecommons.org/licenses/by/4.0/

REVIEWER COMMENTS

Reviewer #1 (Remarks to the Author):

This paper reports a very impressive dataset of coupled LiDAR and forest inventory data from two networks of large forest plots spanning latitudinal transects through Asia and the Americas. This dataset is coupled with climate variables derived from gridded products to examine drivers of spatial variation in productivity and, in particular, to investigate the role of canopy structure in affecting productivity.

Improving our capacity to understand the ecological significance of remotely sensed metrics of forest ecosystems is very important and timely in the face of global change and the need for large-scale ecological monitoring. This paper presents a dataset with substantial potential to generate critical insights. I very much appreciated the mammoth scope of the project and objectives of this study.

I have a few general concerns or queries about the manuscript along with a series of minor comments or suggestions, which I outline below. I hope the authors will find these comments helpful in revising their manuscript.

We sincerely thank you for your constructive comments, which has been greatly strengthened our manuscript. We have undertaken a thorough revision to address all your concerns, with the key improvements outlined below.

1) Reframing the Narrative and Conclusions: We have reframed the manuscript around "broad spatial gradients" and "latitudinal patterns," removing overstated claims about temporal climate change.
2) Accounting for Key Drivers: We have formally incorporated natural disturbance history into our models, improving explained variance (e.g., productivity R^2 increased from 54% to 59%), while acknowledging soil data limitations in the Discussion.
3) Enhancing Conceptual Precision: We now clearly define canopy structural diversity as an emergent property of three structural components (height, cover, vertical complexity), with consistent terminology throughout the manuscript.

Hopefully our following point-to-point responses have addressed your concerns.

This study seeks to account for major drivers of forest productivity across a very large area, and considers climate, species richness, topography and canopy structure. One

question that deserves some careful thought is whether there are other key drivers of productivity that need to be accounted for. Two main thoughts come to mind: soil fertility, and disturbance. Variation in soil fertility among plots (or within plots) does not seem to have been considered; could the authors please consider and comment on this omission?

We agree that soil fertility and natural disturbance are key drivers of forest productivity, and we appreciate the opportunity to address this point. While we did not have direct, plot-level measurements of soil nutrients of all plots in this study, our analyses have included vegetation (e.g., species diversity and canopy cover), climatic and topographic variables (including convexity as topographic position) that are important factors determining soil nutrients besides parent material, soil age and other soil properties (McBratney et al. 2003). This may partly capture the edaphic effect on productivity by those indirect factors.

We fully acknowledge that the omission of soil fertility data is a limitation of our study. As the reviewer correctly points out, variation in edaphic conditions could explain some of the residual variance in our models. We have now added the following paragraph (Lines 213-218) to the discussion to explicitly address this limitation. We discuss how soil fertility likely co-varies with some of our included predictors (e.g., climate and topography).

"Compounding this mechanistic challenge is a second, data-driven limitation: the omission of soil fertility due to a lack of consistent, plot-level data across our study area. Variation in soil properties (e.g., nutrient availability, organic matter) likely contributes to unexplained variance in our models, though some of its effect may be indirectly captured by climate and topography, with which soil development is closely linked." (lines 213-218)

McBratney, A. B., M. L. Mendonça Santos, and B. Minasny. 2003. On digital soil mapping. Geoderma 117:3-52.

On disturbance and accounting for forest age or developmental stage, it is clear that the forests have not been subject to strong recent anthropogenic disturbance (line 212). However, it would be helpful to consider the extent to which natural disturbances, such as fires and hurricanes, have affected the forests. For instance, I understand that the Luquillo plot, at least, has experienced substantial hurricane disturbance. I would expect that disturbance events (or regimes) and the resulting age or developmental stage

of the stands might be expected to strongly influence both forest structure and productivity. If possible, it would be helpful to account for this both in the conceptual reasoning as well as formally in the statistical models.

Thank you for this insightful and constructive comment. You are absolutely correct that natural disturbance regimes, such as hurricanes in the Luquillo forest, are a critical driver of forest structural diversity and productivity. We agree that accounting for this will significantly strengthen our manuscript. We have addressed your suggestion in the following two ways:

1. we have expanded our conceptual introduction in the manuscript, added natural disturbance as a driver of species diversity, canopy structural diversity and forest productivity as in Figure 1 (Lines 568-572):

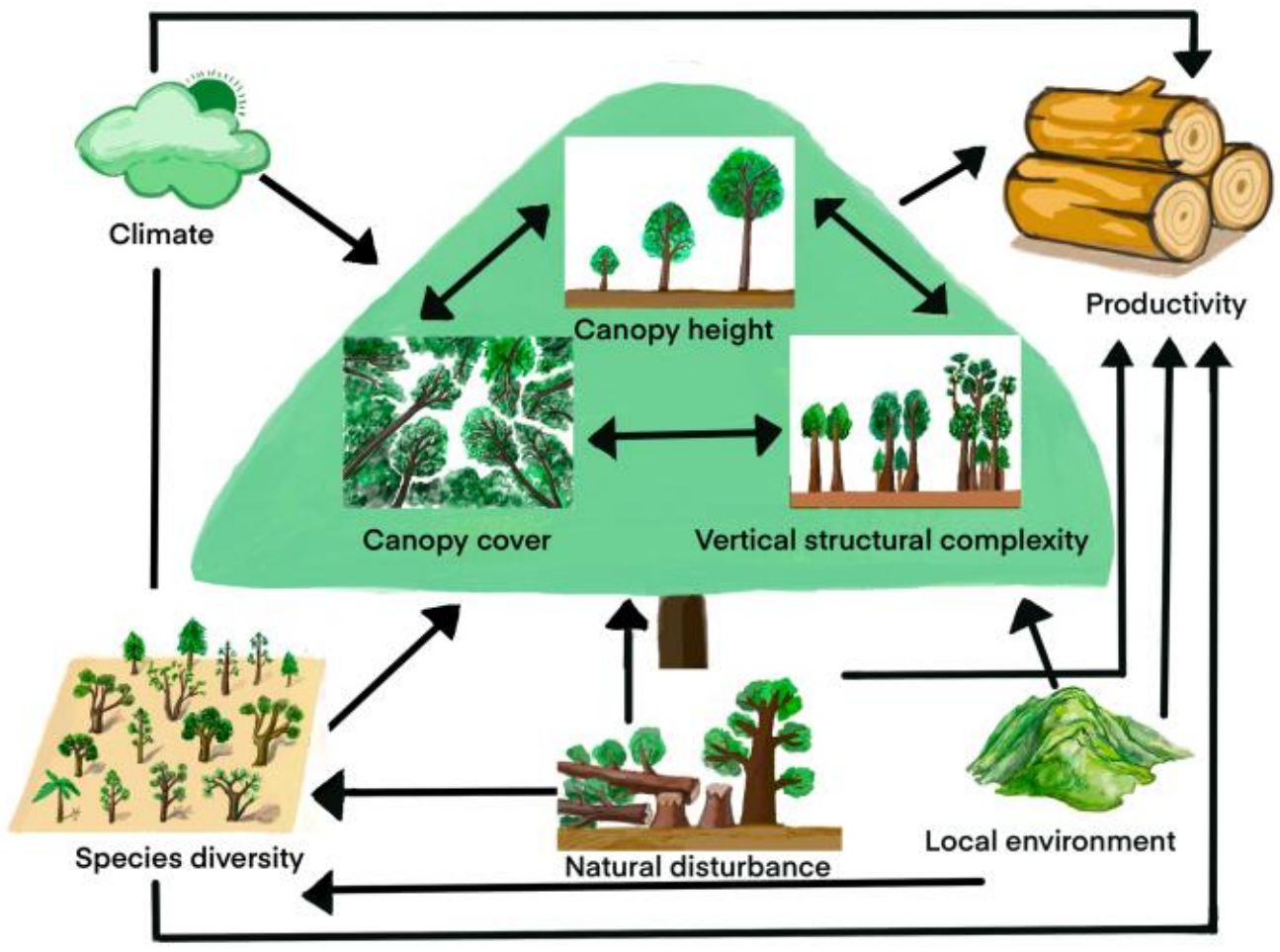

Fig. 1. Conceptual illustration of direct and indirect drivers of forest productivity. Piecewise structural equational modelling (SEM) accounting for direct and indirect effects of climate, species diversity (quantified as species richness), components of canopy structural diversity, natural disturbance and local environment (e.g. topography) on forest productivity. This conceptual illustration for drivers of forest productivity was created by Xiangtao Su.

2. Following your suggestion, we employed three metrics, mean individual basal area per quadrat, skewness of DBH class distribution and net annual biomass change, to approximate the effect of natural disturbance on productivity in Methods section (Lines 352-367) as following:

"Effect of natural disturbance Natural disturbances at stand scale (e.g. from fire, wind, or disease) can create canopy gaps, promoting the growth of smaller trees and shifting stand size structure towards younger, smaller individuals. To evaluate these

effects, we approximated disturbance history using three metrics: 1) mean individual basal area per quadrat as a proxy of development stage, 2) the skewness of DBH class distribution using 5 cm intervals, where a positive skew indicates a regenerating stand, and 3) net annual biomass change (calculated as biomass gain from growth and recruitment minus loss from mortality), where high net increases suggest recovery and net decreases signal recent disturbance. Biomass was estimated using the methods described in "Above-ground productivity and species diversity" section. As these metrics were not directly comparable across plots, each was standardized to unit variance within its respective plot. While all three metrics showed significant but weak correlations with productivity (marginal R^2=0.01~0.07, $p<0.001$), we selected net annual biomass change as our representative measure because it directly quantifies the functional response of the ecosystem to disturbance by integrating the net effects of growth, recruitment, and mortality on carbon dynamics."

Then we have formally incorporated net annual biomass change as a proxy of natural disturbance into our piecewise structural equation models to account for the variation you highlighted. The results of the updated models are presented in Figure 3, and the 'Methods' section has been updated accordingly (Lines 352-367). Its inclusion did not qualitatively change our main conclusions but the updated models explained a greater portion of the variance in forest productivity (former 54% vs current 59%), species richness (54% vs 54%) and canopy structure (canopy cover 32% vs 33%, canopy height 43% vs 44%, vertical structural complexity 39% vs 39%) as shown in Figure 4.

I found some of the conceptual framing and conclusions did not appear to be fully supported by study. For example, the study is framed in terms of climate change. This choice seemed slightly strange to me. While I agree that the insights into forest ecosystem change under climate change are critical, the study does not investigate climate change and instead looks at spatial variation over broad climatic/latitudinal gradients. This line of interpretation is followed through to the conclusions of the paper, with a major finding reported to be that dynamics of forest structure will be capture climate change effects on productivity. From the results and reasoning provided, I was not entirely convinced that the observed spatial relationships with climate will necessarily reflect changes over time at a given location. Given the potential consequences of these conclusions (i.e., that these findings might inform policy and direct the allocation of monitoring efforts), I believe it is very important that these

findings are robust and well-substantiated.

Similarly, in both the abstract and final paragraph in the discussion, there is a statement to the effect that the results emphasise 'the importance of incorporating canopy structural diversity into conservation priority frameworks'. I found this conclusion somewhat unconvincing or open to misinterpretation. How do these results connect directly with conservation objectives? I believe these statements ought to be tempered or the connections much more clearly explained.

We sincerely thank the reviewer for this critical insight. We agree that our original framing overstated the study's direct relevance to *temporal* climate change impacts, given our spatial-gradient design. We have now comprehensively revised the manuscript to:

1. **Reframe the Introduction and discussion sections around spatial gradients:**

   Replaced *"climate change"* with **"broad spatial gradients"** (e.g., Abstract, Introduction, Discussion), while we removed all language implying temporal dynamics (e.g., *"restructuring," "future climate change", "over time"*).

   In Abstract section:

   "Climate change is restructuring forest canopies through species turnover and altered three-dimensional complexity across boreal, temperate, and tropical biomes. Although these structural shifts are globally pervasive, their consequences for forest productivity remain unresolved—particularly how climate-mediated shifts in canopy structural diversity may modulate direct physiological constraints from changing climatic regimes." (Lines 4-9 in former Abstract)

   This narrative was associated with temporal climate change. To stress variation in structural diversity and productivity along broad spatial scale, now we revised this sentence as "Canopy structural diversity varies systematically with climate and species diversity across boreal, temperate, and tropical biomes. Yet, how this latitudinal variation in canopy structural diversity affects forest productivity—particularly the role of structural diversity in mediating effects of climate on forest across biomes—remains unresolved." (Lines 4-7)

For Introduction section, we had similar revision:

Lines 28-34 in former Introduction: "Under projected warmer and drier climates, rising drought stress and disturbance frequency are projected to reduce canopy height, cover, and complexity, favoring smaller trees with concomitantly diminished leaf area. Resolving how these structural diversity declines will propagate to productivity losses requires disentangling the dual roles of climate: as a *direct* physiological constraint on tree growth and an *indirect* architectural mediator via structural diversity."

We revised these sentences as:

"The strength of this relationship, however, may be modulated by climate via two pathways: directly, through physiological constraints on individual tree growth, and indirectly, by altering structural diversity that determines light-interception and use efficiency. Resolving the mechanistic link between these climate-driven shifts in structural diversity and observed productivity patterns across spatial gradient thus requires disentangling the dual roles of climate: its direct physiological constraints on growth versus its indirect effects on productivity mediated by structural diversity." (Lines 27-34)

For Discussion section:

"Our results support our first prediction that in addition to the direct effects, there is an indirect pathway by which climate affects productivity: via shifts in canopy structural diversity—a pathway that is overlooked in current ecosystem models. These indirect effects arise from latitudinal gradients in climate and species richness that shape canopy structural diversity via physiological constraints on tree height, canopy cover and vertical complexity." (Lines 129-134)

We stress spatial patterns across broad latitudinal and climatic gradients in the revised version as following:

"We are able to go beyond this direct effect, however, and support our first prediction that climate indirectly affects productivity across biomes via shifts in canopy structural diversity (e.g. crown complementarity, three-dimensional spatial heterogeneity). This mechanistic pathway is not represented in many dynamic global vegetation models, which characterize forest canopies using large-scale mean structural metrics (e.g., leaf area index and height) without accounting for structural diversity. In our study, across broad latitudinal and climatic gradients, covariation between climate and species richness appears to shape structural diversity via

physiological constraints on tree height, canopy cover, and vertical complexity" (Lines 145-153)

2. **Reposition policy implications:**

Revised conservation claims to focus on current forest functionality:

We deleted this sentence "We propose leveraging satellite-based LiDAR to track canopy structural diversity dynamics—a scalable approach for monitoring global forest productivity responses to climate change and biodiversity loss." (Line 197-199 in the former Discussion)

We also revised previous policy implication "We identify a consistent spatial correspondence between hotspots of canopy structural diversity and productivity, suggesting that conservation frameworks could prioritize structural diversity as a key metric." as "We identify a consistent spatial correspondence between hotspots of canopy structural diversity and productivity, suggesting that monitoring canopy structural diversity could enhance assessments of forest productivity." (Lines 220-223)

3. **Adjust conclusion:**

We adjust conclusion "we emphasize the necessity of integrating climate-driven changes in canopy structural diversity into ecosystem models to refine predictions of carbon sequestration and forest resilience. These insights bridge mechanistic understanding with actionable strategies for preserving forest resilience in an era of global climate change." as "integrating climate-driven changes in structural diversity into ecosystem models may improve predictions of carbon sequestration, providing a more mechanistic understanding of forest responses to climate change." (Line 226-228)

A broader issue on framing: Canopy height, canopy cover and vertical structural complexity are presented in the introduction (lines 24-25) as measures of structural diversity. This is an issue I have seen elsewhere in the literature as well. I would argue that the latter captures a dimension of heterogeneity, but the first two might be more accurately described simply as measures of forest structure.

We thank the reviewer for this important clarification regarding the distinction between structural attributes and structural diversity. We agree that canopy height and canopy cover are fundamental attributes of stand-level structure, while vertical complexity explicitly captures heterogeneity at finer scales. In our revision, we have clarified this

distinction and now explicitly define canopy structural diversity as the three-dimensional heterogeneity in volume and arrangement of above-ground vegetation (e.g., leaves and branches)–is an emergent property arising from three components: canopy height, canopy cover, and vertical complexity (i.e., canopy depth and leaf area distribution), following the definition of canopy structure form Fahey RT, et al. (2019) and Valbuena R, et al. (2020).

Previous description of canopy structural diversity in the Introduction:
This structural diversity, defined as the three-dimensional heterogeneity in canopy volume and arrangement, is characterized by three interdependent components: canopy height, canopy cover, and vertical complexity (i.e., canopy depth and leaf area distribution) (Lines 22-25 in former Introduction)
Current description of canopy structure in the Introduction:
The canopy structural diversity－the three-dimensional heterogeneity in volume and arrangement of above-ground vegetation (e.g., leaves and branches)－is an emergent property arising from three components: canopy height, canopy cover, and vertical complexity (i.e., canopy depth and leaf area distribution), (Lines 21-25).
Fahey RT*, et al.* Defining a spectrum of integrative trait-based vegetation canopy structural types. *Ecol. Lett.* **22**, 2049-2059 (2019).
Valbuena R*, et al.* Standardizing Ecosystem Morphological Traits from 3D Information Sources. *Trends Ecol. Evol.* **35**, 656-667 (2020).

On one level, the writing is quite smooth. However, on closer reading, I found some of the writing and logic unclear. In general, precision and specificity could be improved. There are instances where unnecessary words are added that increase complexity and potentially cause confusion without aiding meaning. There is also some inconsistency in the terms given to metrics. Overall, these issues obscure the potential impact of the manuscript. I have pointed out some examples in my specific comments (below) and would recommend carefully revising the entire manuscript.

Minor comments
Line 9: In this context, the intended meaning of 'inter-calibrated' was unclear to me. Is this word necessary? If so, could it perhaps be further defined in the main text?

Response: This word is not necessary, and we delete this word in the new version of our manuscript.

Line 11: I presume beyond their direct effects on 'forest productivity'.

Response: Following your suggestion, we revise the former sentence "we show that beyond their direct effects, climate and species diversity influence above-ground forest productivity indirectly through regulating canopy structural diversity." as "we show that beyond its direct effects on forest productivity, climate influence productivity indirectly by regulating canopy structural complexity—a significant, yet previously underappreciated mechanism, whose precise magnitude is challenging to isolate." (Lines 9-12)

Line 21-22: I think I follow the intended meaning, but this phrasing seems somewhat imprecise to me.

Response: Following your suggestion, we revised the former sentence "Forest canopies, the collection of tree crowns in a forest stand, capture up to 98% of incoming solar energy in dense forests, positioning their structural diversity as a critical nexus for ecosystem energy capture." as "Forest canopies—the collective layer of tree crowns in a stand—capture up to 98% of incoming solar energy in dense forests, highlighting the role of canopy structural diversity in driving ecosystem energy capture."(Line 19-21)

Line 34: This sentence is unclear (architectural mediator of what?).

Response: Here we mean that architecture is a mediator of the effects of climate on productivity. Following your suggestion, we revised previous sentence "Resolving how these structural diversity declines will propagate to productivity losses requires disentangling the dual roles of climate: as a *direct* physiological constraint on tree growth and an *indirect* architectural mediator via structural diversity" as "Resolving the mechanistic link between these climate-driven shifts in structural diversity and observed productivity patterns across spatial gradient thus requires disentangling the dual roles of climate: its direct physiological constraints on growth versus its indirect effects on productivity mediated by structural diversity." (Lines 31-34)

Line 45: While plausible, this statement appears unsubstantiated. Is this intended as a hypothesis or as a substantiated statement?

Response: The relationship between species richness and canopy structural diversity as well as the relationship between functional diversity and canopy structural diversity has been confirmed by a few observational studies (e.g. Coverdale and Davies. 2023, Journal of Ecology) and manipulative experiments (e.g. Ray et al. 2023, Science Advances). Despite of this evidence, the reviewer is correct to note that this is a plausible mechanistic explanation rather than a directly substantiated finding from our present study. The original statement has been tempered by adding the word 'potentially': We revised the former sentence as "warmer and wetter climates support species-rich communities with diverse functional strategies that potentially enhance vertical and horizontal complementarity in crown architectures" (Lines 42-44)

Coverdale, T. C., and A. B. Davies. 2023. Unravelling the relationship between plant diversity and vegetation structural complexity: A review and theoretical framework. Journal of Ecology 111:1378-1395.

Ray, T., B. M. Delory, R. Beugnon, H. Bruelheide, S. Cesarz, N. Eisenhauer, O. Ferlian, J. Quosh, G. von Oheimb, and A. Fichtner. 2023. Tree diversity increases productivity through enhancing structural complexity across mycorrhizal types. Science advances 9:eadi2362.

Line 46: In what sense are these mechanisms synergistic?

By 'synergistic,' we mean that the mechanisms – primarily (1) the creation of heterogeneous light environments by heterogeneous crown morphologies and (2) the utilization of these light environments by coexisting species with diverse functional traits (leaf types) – interact in a way that produces a greater increase in productivity than either mechanism would generate alone. In the revised version, we also used "synergy" to express the interaction between direct and indirect effects of climate on productivity. We revised former sentence "These mechanisms synergistically drive higher structural diversity at lower latitudes, where heterogeneous light regimes create opportunities for partitioning among coexisting species with diverse leaf types and crown morphologies" to clarify this interaction as "The synergy between climatic effects intensifies toward the tropics, thereby strengthening the relationship between structural diversity and forest productivity across latitudinal gradients. Specifically, relaxed physiological constraints interact with heterogeneous crown morphologies to

create distinct light niches that are exploited by coexisting species with diverse leaf types, driving higher productivity." (Lines 44-49).

Line 47: Partitioning of what?

This sentence was revised and we hope this problem no longer exists.

Line 72: Two quadrat sizes are mentioned here and again in the methods section. However, it was not clear to me why two quadrat sizes were chosen and, indeed, how the two quadrat sizes were used. All analyses appear to use the 50 m x 50 m quadrats.

Two quadrat sizes 50 m × 50 m and 100 m × 100 m were chosen in this study to avoid the border effect, as tree crowns are not constrained by plot borders. In a simulation study, (Knapp et al. (2021) found that the border effects accounted for 40% of the biomass estimation uncertainty at the 20 m × 20 m scale, but had no contribution at the 100 m × 100 m scale. This was also confirmed by Cushman et al. (2023). We thus chose quadrat sizes 50 m × 50 m and 100 m × 100 m to avoid border effects. This was clarified "Each forest plot was divided into non-overlapping 50 m × 50 m (49-200 per plot) and 100 m × 100 m (6-50 per plot) quadrats to balance the trade-off between minimizing the uncertainty in biomass estimation from border effects and maintaining sufficient sample size for robust cross-plot statistical analysis (Supplementary Table 1). This design mitigates the ~40% uncertainty from border effects at a 20 m × 20 m scale while ensuring sufficient sample size." between Lines 253-258 in Methods section. The results from the 100 m × 100 m quadrat size were similar to those from the 50 m × 50 m size, thus only results for the 50 m × 50 m quadrat are presented.

Knapp, N., A. Huth, and R. Fischer. 2021. Tree crowns cause border effects in area-based biomass estimations from remote sensing. Remote Sensing 13:1592.

Cushman, K. C., S. Saatchi, R. E. McRoberts, K. J. Anderson-Teixeira, N. A. Bourg, B. Chapman, S. M. McMahon, and C. Mulverhill. 2023. Small Field Plots Can Cause Substantial Uncertainty in Gridded Aboveground Biomass Products from Airborne Lidar Data. Remote Sensing 15:3509

Line 129-131: This statement was unclear to me. Canopy height and cover are modelled in many DGVMs, for instance. It would help to be more specific about the kinds of ecosystem models and the kinds of metrics that are being referred to here.

Thank you for this important clarification. We agree that canopy height and cover *are* modelled in many DGVMs and similar models. Our point was that **canopy structural diversity**—specifically, the heterogeneity in crown architectures, i.e., vertical complexity, and spatial arrangement of crowns—is typically not explicitly modeled as a *mechanistic pathway mediating climate-productivity relationships*. Many models are structured around mean values of large-scale parameters (e.g., total leaf area index, mean canopy height) rather than resolving how *structural diversity* emerges from climate and in turn drives productivity. We will revise the text to clarify:

(1) the *specific aspects* of structural diversity unrepresented (e.g., crown complementarity, 3D heterogeneity),

(2) that we refer to its role as a *causal intermediary pathway*.

We revised the former sentence "Our results support our first prediction that in addition to the direct effects, there is an indirect pathway by which climate affects productivity: via shifts in canopy structural diversity—a pathway that is overlooked in current ecosystem models." to "We were able to go beyond this direct effect, however, and found strong support for our first prediction climate indirectly affects productivity across biomes via shifts in canopy structural diversity (e.g. crown complementarity, three-dimensional spatial heterogeneity). This mechanistic pathway is largely unrepresented in many dynamic global vegetation models, which characterize forest canopies using large-scale mean structural metrics (e.g., leaf area index and height) without accounting for structural diversity" (Lines 145-151).

Line 151-153: As for the previous comment, some clarification or greater specificity about the additional processes being proposed would be helpful. It is my understanding that models already exist that include some of these processes by which environmental conditions limit, for instance, the height and density of forest canopies.

Thank you for highlighting this ambiguity. We agree that canopy height and cover *are* represented in DGVMs. Our critique focuses on how **canopy structural complexity**—such as *crown complementarity, vertical stratification heterogeneity, and 3D spatial arrangement*—functions as a **mediator** in climate-productivity feedbacks. Many current DGVM implementations:

- Represent structure via **large-scale mean metrics** (leaf area index, height) rather than resolving canopy structural complexity.

- Treat structure as a *static outcome* rather than modeling its *causal role* in enhancing productivity (e.g., via niche partitioning).

We will revise the text to clarify:

(1) the *specific structural metrics* overlooked (beyond large-scale mean height/cover),

(2) the *mechanistic gap* in modeling diversity-driven feedbacks,

(3) that our call targets **next-generation DGVMs** seeking to capture structural diversity-mediated ecosystem processes.

We revised the former sentence "We suggest that dynamic global vegetation models must integrate these climate-structural diversity-productivity feedbacks to improve predictions of forest responses to future climate change scenarios." to "Integrating climate-structural diversity-productivity feedbacks into next-generation dynamic global vegetation models—moving beyond mean structural metrics toward more mechanistic representations of structural diversity—will improve risk assessment for ecosystem services and inform climate-adaptive forest management." (Lines 167-171)

Line 176: What is structural complementarity?

Thanks for highlighting this ambiguity. Spatial complementarity of canopies is an ecological concept describing how different plant species within a forest community arrange their leaves and branches in three-dimensional space in such a way that they minimize direct competition for light while maximizing the overall capture of sunlight by the community as a whole. In the context of this sentence, elevated functional diversity (e.g. larger range of shade-tolerance) in tropical forest could enhance the spatial complementarity of canopy arrangement.

To clarify, we revised the former sentence "Large-scale patterns include elevated functional diversity which may enhance structural complementarity" to "Large-scale patterns that could contribute include elevated functional diversity which may enhance *canopy spatial complementarity* among coexisting species" (Lines 194-196).

Line 197-199: I am not sure about this conclusion. This study examines change over space and not change over time. It is not clear to me that these patterns would necessarily be substitutable.

As our study focus on variation of canopy structural diversity along spatial gradients rather than temporal changes in canopy structural diversity, we agree that this wording is not appropriate and we deleted this sentence.

Methods

Line 217: I appreciate these tables, but it takes some effort to compare the timing of the forest inventories with the LiDAR measurements. I would recommend keeping the tables. However, would it be possible to also add a summary to the main text of how closely the two datasets are matched in time? It appears that most (but not all) overlap in time, with some LiDAR measurements occurring toward the beginning and others toward the end of the census interval.

We thank the reviewer for this helpful suggestion regarding the temporal alignment between the LiDAR acquisitions and forest census periods. We have now added a summary in the Materials and Methods Section/Study Sites, explicitly summarizing the temporal relationship between the LiDAR survey dates and the forest census intervals across all 16 plots. This summary quantitatively describes the timing (e.g., LiDAR within census, before start, after end) and the degree of overlap for each category. The detailed table remains essential for transparency of site-specific parameters, but the new text provides the requested high-level overview of the temporal matching. We added as the following summary: "LiDAR acquisition occurred within the census interval for 9 plots, after the census ended for 6 plots (Luquillo, Gutianshan, Donglingshan, Tiantong, Fenglin and Daxinganling), and before the census began for 1 plot (Barro Colorado Island). Despite this variation, LiDAR data capture canopy structure typically within 0-5 years of the measured census period for all plots, supporting robust links between canopy structural diversity and forest productivity." (Lines 2244-249).

Line 221: The phrasing here was slightly unclear me: smaller than what? I think this is simply referring to small plots.

Thanks for highlighting this ambiguity. As we mentioned in response to a previous comment, small quadrats (e.g. 20 m × 20 m quadrat) suffer from border effects that accounted for 40% of the biomass estimation uncertainty at the 20 m × 20 m scale, but have no contribution for large quadrat (e.g., 100 m × 100 m scale). So we revised the text as suggested.

Line 223: Would it be possible to clarify how many quadrats were at each plot? Perhaps a range could be added to the main text and this information also added to a new column in Supplementary Table 1.

In general, it would also help to clearly explain the spatial scale of analyses (i.e., plot or quadrat or both) and why this spatial scale was chosen or appropriate for the given analysis.

We thank the reviewer for highlighting the need for greater clarity regarding our choice of spatial scale. We have revised the text (Section [Mention Section, e.g., Methods / Data Processing]) to explicitly state the rationale for analyzing biomass at the 50 m × 50 m and 100 m × 100 m quadrat scales. This decision was driven by two key factors: (1) minimizing uncertainty from border effects in biomass estimation, which studies show become negligible at the 100 m scale (Knapp et al. 2021, Cushman et al. 2023), and (2) ensuring a sufficient number of quadrats per plot for robust statistical analysis, which would be compromised at larger scales. The original table listing quadrat counts per plot (Supplementary Table 1) further supports point (2). We revised the former sentence to "Each forest plot was divided into non-overlapping 50 m × 50 m (49-200 per plot) and 100 m × 100 m (6-50 per plot) quadrats to balance the trade-off between minimizing the uncertainty in biomass estimation from border effects and maintaining sufficient sample size for robust cross-plot statistical analysis (Supplementary Table 1). This design mitigates the ~40% uncertainty from border effects at a 20 m × 20 m scale while ensuring sufficient sample size." (Lines 253-258)

Line 240: Would it be possible to add some more details here: how closely is "closely aligned"?

Their correlation coefficient is $r=0.89$, $p<0.001$, and we added this to the Methods section (Lines 277-278).

Line 244-248: It would help to explain the reasoning behind this analysis; as it stands, it is not clear (e.g. what sampling bias exactly?). Also, more broadly, the rationale for the beta-diversity analysis was not well introduced.

We agree the need of greater clarity regarding the rationale for our beta-diversity analysis and the specific sampling bias addressed by the null model. We have revised the text to explicitly state: (1) the ecological motivation for examining the effect of beta-diversity on productivity (species identity effects), and (2) the specific sampling bias controlled by the null model (inflated beta-diversity expectations in regions with higher gamma-diversity due purely to sampling effects from larger species pools). We cite Myers et al. (2013) as the methodological citation for this null model approach and

clarify that our standardized residuals represent beta-diversity beyond the sampling effect. We thus revised the former description of the beta-diversity analysis method:

"To quantify the effect of β-diversity (species compositional differences among communities) on productivity, we performed principal coordinates analysis on a Bray–Curtis dissimilarity matrix derived from species abundance data across quadrats within each plot. To control the sampling bias, we standardized β-diversity using a spatial null model that randomized individual tree locations while maintaining plot-level species richness, stem density and species abundance distributions. Observed β-diversity values were standardized as residuals relative to the null expectation (mean of 1,000 randomizations) using the '*vegan*' R package." to "To quantify effects of unique quadrat compositions (e.g., dominance of fast-growing species), we analyzed how β-diversity relates to productivity within each plot. We calculated β-diversity using a Bray–Curtis dissimilarity matrix derived from species abundance data across all quadrats. This matrix was then summarized via principal coordinates analysis (PCoA) to represent the compositional uniqueness of each quadrat by its position in PCoA space (e.g., distance to the plot centroid based on the selected axes). Because observed β-diversity tends to increase with γ-diversity due to sampling effects: larger species pools make local communities share fewer species by chance. To isolate biologically meaningful compositional variation from this sampling effect, we standardized β-diversity using a spatial null model. The model randomized individual tree locations within each plot while preserving plot-level species richness, stem density, and species abundance distributions (thereby fixing γ-diversity). We computed the mean of the PCoA-summarized Bray–Curtis dissimilarities from 1,000 randomizations as the null expectation. Using the 'vegan' R package, we then computed residuals of observed β-diversity relative to the null expectation, quantifying β-diversity exceeding sampling effects." (Lines 279-294)

Hopefully the beta-diversity analysis method is clear now.

Line 249: Was the entire area of each plot surveyed? This is currently not clear.

We added a column in Supplementary Table 4 to indicate Scanning area/total area in ha. Hopefully it is clear now.

Line 264: Here and among the figures, there appears some inconsistency in the terminology being used for different metrics (e.g., vertical structural complexity,

foliage height diversity; canopy height, mean outer canopy height). It is unclear if these are the same or different metrics, and it would help for this to be presented very clearly and consistently throughout.

We thank the reviewer for identifying inconsistencies in our terminology for LiDAR metrics. We have revised the text and figures to use the following **terminology** consistently:

- **Canopy height** (general term) → quantified as **mean outer canopy height** (specific metric)
- **Canopy cover** (general term) → quantified via **first-return cover above 2m** (method)
- **Vertical structural complexity** (general term) → quantified as **foliage height diversity** (specific metric).

We revised the former paragraph: "For each 50 m × 50 m quadrat, we quantified canopy height, horizontal cover and vertical structural complexity from first LiDAR returns, which is fairly robust to variations in flight altitude and footprint size. Mean outer canopy height was calculated as the average height of first returns, assuming that first returns accurately represent the outer canopy surface due to limited penetration into vegetation layers. Canopy cover was quantified as the proportion of the number of first returns above 2 m relative to the total number of first returns, while foliage height diversity was calculated to quantify vertical structural complexity, applying the Shannon-Weiner diversity function applied to the vertical distribution of first return heights. Foliage height diversity is expected to increase with greater canopy height extent and evenness in foliage layering. All LiDAR metrics were calculated using the 'lidR' package"

The revised paragraph explicitly defines this relationship between general concepts and specific metrics as "For each 50 m × 50 m quadrat, we quantified canopy height, canopy cover and vertical complexity from first LiDAR returns, which are robust to variations in flight altitude and footprint size. Canopy height was calculated as the average height of first returns (i.e., mean outer canopy height), assuming that first returns accurately represent the outer canopy surface due to limited penetration into vegetation layers. Canopy cover was quantified as the proportion of the number of first returns above 2 m relative to the total number of first returns, while vertical

complexity was quantified as foliage height diversity, calculated by applying the Shannon-Weiner diversity function to the vertical distribution of first return heights. Vertical complexity is expected to increase with greater canopy height extent and evenness in foliage layering. All LiDAR metrics were calculated using the 'lidR' package." (Lines 303-313)

We have applied this standardized terminology throughout the manuscript and figures.

Line 296: Could the meaning of 'altitude above channel' be clarified?

We clarified the former definition "altitude above channel was represented as the vertical distance from the channel network" as "altitude above channel was measured as the vertical distance above the nearest water channel in the valley" (Lines 348-349). Hopefully it is clear now.

Line 314: Here I was slightly confused about the model selection. This seems to imply that multiple models were retained, and it is unclear how they were subsequently used.

The sentence was revised to explicitly state that all models within $\Delta AIC \leq 2$ were retained. Subsequently, individual and combined climatic variables in these retained models were identified as key climatic determinants. This approach is standard practice when multiple models show similar empirical support (Burnham & Anderson, 2004). The Methods section now clearly describes this workflow. This sentence "Model selection was performed using Akaike Information Criterion (AIC), retaining the models within $\Delta AIC \leq 2$ from the optimal model." was revised to "Model selection was performed using the Akaike Information Criterion (AIC), retaining all models within $\Delta AIC \leq 2$ from the optimal model. The climatic variables featured in these retained models were identified as key determinants." (Lines 374-377)

Line 335: Please explain why and how the topographic variables were combined into a composite variable.

We agree that the rationale and methodology for combining these variables should be explicitly stated. As suggested, we have revised the manuscript to explain both why (to account for combined topographic effects on productivity) and how (using a rigorous statistical approach) the composite variable was derived. Specifically, we revised the former sentences "We also included a composite variable "Topography", derived from

convexity, slope and the topographic wetness index, as these significantly influenced productivity in preliminary analyses" to: "To represent the collective influence of topography, we also created a composite variable "Topography". Key topographic variables were first identified through AIC-based model selection on multiple linear regressions with productivity. As convexity, slope, and topographic wetness index consistently improved model performance, the "Topography" composite variable was constructed as a weighted linear combination, using the standardized coefficients from the best AIC-selected regression model as weights." (Lines 398-404)

The weights for the linear combination were derived from standardized regression coefficients in the optimal model, ensuring the composite variable reflects the relative influence of each topographic factor on productivity. This approach allows us to efficiently capture the combined effect of topographic variables while reducing multicollinearity concerns.

Line 340: Please explain why these transformations were appropriate.

Thank you for requesting clarification. We have revised the manuscript to explicitly justify each transformation based on statistical assumptions of SEM and mixed models:

1) Log-transformation of productivity:

Addressed heteroskedasticity (variance in productivity increased with biodiversity levels, e.g., higher diversity plots showed greater variability).

Linearized the saturating biodiversity-productivity relationship (diminishing returns at high diversity), as the log-transform better captures non-linear ecological scaling.

2) Logit-transformation of canopy cover:

Corrected inherent variance instability in proportions: variance peaks at 0.5 cover and decreases near 0 and 1 bounds. The logit transform stabilizes variance across the data range.

3) Standardization (scaling to unit variance) facilitates comparison of effect sizes (SEM path coefficients, mixed model fixed effects) by eliminating unit-of-measurement differences.

For simplicity, we revised the former sentence "Prior to analyses, we log-transformed productivity, logit-transformed canopy cover, and standardized all variables to unit variance." as "Prior to analyses, we log-transformed productivity and logit-transformed canopy cover to mitigate heteroskedasticity, and standardized all variables to unit

variance for direct effect size comparison in SEM models." (411-413)

Fig. 3: Please define the abbreviation CWP

In previous version of manuscript, we used coarse wood production (CWP) to represent forest production. In this version, we used above-ground productivity to measure forest productivity. So we hope that the problem does not exist now.

Fig. 4: It seems common to emphasise whether relationships are positive or negative in structural equation models. However, that information is not emphasised here, and I wondered about that decision.

We agree that explicitly stating directionality strengthens the interpretation of SEM pathways. We have revised the paragraph to emphasize the **positive direction** of all key relationships while retaining our original conclusions about effect magnitudes. We revised the original paragraph to "Climate and species richness positively regulated productivity both directly and indirectly through their positive effects on components of structural diversity (Fig. 4a, b). Climate had a stronger positive effect on productivity than species richness or local topography alone. In addition, the combined influence of components of structural diversity collectively had a greater positive effect on productivity than either climate or species richness alone (Fig. 4b). Structural diversity ~~was~~ remained positively correlated with productivity, even after controlling for climate, species richness and local topography (Fig. 4a)." (Lines 128-135)

Supplementary Table 4: I see there are two LiDAR surveys of Barro Colorado Island. Perhaps I missed it, but it was unclear to me if and how the two surveys were used (i.e., were both surveys used and treated as separate datapoints, or was one chosen, or were the two surveys somehow combined?). Could this please be clarified?

Thank you for your careful review of Supplementary Table 4. We apologize for the ambiguity in the table formatting – there was only one LiDAR survey conducted for Barro Colorado Island. The two rows of sensor and date inadvertently suggested separate surveys when they were intended to list complementary metadata for the same survey:

- Sensor: Optech ALTM Gemini;
- Survey Date: August–September 2009.

No additional surveys exist, and all analyses used this single 2009 dataset.

we Revised **Supplementary Table 4** to list sensor and survey date in a single row.

Reviewer #2 (Remarks to the Author):

This is a unique study that reassesses and rethinks the impact of climate change on forest productivity. In contrast to conventional approaches. The authors examine not only the direct effects of factors such as mean annual precipitation and mean annual temperature on biomass growth, but also the indirect effects of these factors through related forest characteristics such as species diversity, mean canopy height, canopy cover or leaf height diversity.

Using an exceptional dataset of plots from boreal to tropical forests, including savannas and scrubland, the authors are able to confirm their hypothesis that climate, species diversity and canopy structural diversity together drive the latitudinal gradient in forest productivity.

The data include inventory and airborne laser scanning data collected at 16 study sites between 2005 and 2020. Although the period of data collection differs between study sites, the authors have taken care to ensure comparability of the data, for example by ensuring that all data were collected during the growing season.

The data were analysed using a number of well-chosen statistical models, including linear mixed-effects models, generalised least squares models or stepwise structural equation models. The results clearly show that the indirect effects of climate on canopy structure diversity are significant in shaping forest productivity. The results support the conclusion that this effect should be taken into account when assessing forest responses to climate change, e.g. by including it in global vegetation models.

Overall, the manuscript is very well written and clear. I have only a few comments and suggestions:

We sincerely thank you for your positive assessment of our work and your thoughtful suggestions, which have helped us improve the methodological rigor and clarity of our manuscript. We have made key improvements as following:

1) Addressing Potential Biome Bias: We agree that uneven plot distribution is a common challenge in large-scale studies. A sensitivity analysis confirmed biome identity was not a significant random effect, supporting the robustness of

our cross-biome conclusions.

2) Methodological Rigor: We have clarified the rationale for using two quadrat sizes, detailed our use of region-specific allometric equations, and now explicitly report both marginal and conditional R^2 values.

3) Interpretation of Effects: We have refined the discussion on the direct versus indirect climate effects, acknowledging the challenge in quantifying their precise magnitudes while justifying the qualitative validity of the mediation pathway through independent evidence and consistent latitudinal patterns.

Hopefully the following point-to-point responses have addressed your concerns.

1. Although the dataset is impressive and a result of the available data, it is not very clear to what extent the differences in the number of plots between biomes affect the results. Can you comment on potential biases?

We appreciate this important methodological consideration. The uneven plot distribution reflects data availability constraints in large-scale forest studies but raises valid concerns about biome-level representativeness. To address potential biases, we conducted a sensitive analysis by employing piecewise SEMs with mixed-effects models using plot identity nested within biomes as random effects (model 2), which inherently accounts for unequal sample sizes by partially pooling information across biomes. We compared the piecewise SEM model using plot as a random effect (model1) with model 2 (plot identity nested within biomes) with likelihood ratio test (Supplementary Table 8). We found that biome identity was not a significant random effect as shown in Supplementary Table 8, suggesting that conclusions along the latitudinal gradient across biomes are robustly supported. However, as you pointed out, the number of plots within biomes may affect results along spatial gradient within biomes. Conclusions within tropical rainforests (only 1 plot) should thus be considered exploratory, whereas patterns within temperate forest (7 plots) are supported.

We also added the likelihood ratio test of random effect to the Methods section "Likelihood ratio tests were employed to assess the significance of random effects (biome identity was not significant (Supplementary Table 8) and was excluded from the model), and overall model fit was evaluated using Fisher's C statistic." (Lines 408-411)

2. Line 72: Why do you use different quadrat sizes (50 m x 50 m vs. 100 m x 100 m)?

We thank the reviewer for highlighting the need for greater clarity regarding our choice of spatial scale. Reviewer 1 had a similar concern. We used two different quadrat sizes for (1) minimizing uncertainty from border effects in biomass estimation, which studies show that border effects become negligible at the 100 m scale (Knapp et al. 2021, Cushman et al. 2023), and (2) ensuring a sufficient sample size for robust across-plot statistical analysis, which would be compromised at larger scales. The original table listing quadrat counts per plot (Supplementary Table 1) further supports point (2). We have revised the text [Lines 255-260]) to explicitly state the rationale for analyzing forest productivity at the 50 m × 50 m and 100 m × 100 m quadrat scales. "Each forest plot was divided into non-overlapping 50 m × 50 m (49-200 per plot) and 100 m × 100 m (6-50 per plot) quadrats to balance the trade-off between minimizing the uncertainty in biomass estimation from border effects and maintaining sufficient sample size for robust cross-plot statistical analysis (Supplementary Table 1). This design mitigates the ~40% uncertainty from border effects at a 20 m × 20 m scale while ensuring sufficient sample size."

3. Line 77: It seems that the allometric relations used are always valid for a given species. Do they not change for different sites?

We thank the reviewer for highlighting this important methodological consideration. We acknowledge that allometric relationships vary across sites due to biotic and abiotic factors (e.g., soil, climate). Our approach addressed this issue by using the best available allometric models for each region or biome as follows:

For North American plots, the 'allodb' package incorporates site-specific climatic calibrations to mitigate local variability;

For tropical plots: the 'BIOMASS' package integrates plot-level wood density and height-diameter corrections to account for site effects.

For Asian plots: we used species-specific equations derived from nearby sites with comparable environmental conditions (Supplementary Table 5), minimizing cross-site bias.

We have revised the Methods section to clarify these adaptations:

For temperate ~~and subtropical plots~~ North American plots (Utah, Smithsonian Conservation Biology Institute, Wind River), the aboveground biomass was estimated using ~~taxa-specific allometries in~~ the 'allodb' package, applying taxa-specific

allometries calibrated with site-level climate data to mitigate local variability. ~~while species-specific allometric equations derived from previous studies were employed~~ For temperate and subtropical Asian plots (Ailaoshan, Mulun, Gutianshan, Tiantong, Donglingshan, Changbaishan, Fenglin and Daxinganling), biomass estimates were derived from published species-specific equations developed under similar bioclimatic conditions (see more details in Supplementary Table 5). For tropical plots (Danum Valley, Paracou, Barro Colorado Island, Luquillo and Xishuangbanna), aboveground biomass was calculated using the 'BIOMASS' package was used. ~~Which~~ This package incorporates taxonomic identity and wood density from the Global Wood Density Database and applies local height-diameter allometric corrections to account for site-specific differences." (Lines 265-275)

4. Lines 104 - 106: while it is reasonable to focus on R marginal in the results to explain the fixed effects, R conditional is also presented in Figure 3. It might be good to refer to it and its meaning (fixed + random effect) in at least one sentence.

We appreciate the reviewer's suggestion to provide a more comprehensive interpretation of model performance. We revised the first sentence that mentions R marginal between Lines 99-104 to explicitly define the conditional R^2 (R^2_C) from Supplementary Table 2 and clarify its meaning (variance explained by both fixed and random effects). This addition strengthens the statistical context while maintaining our focus on fixed-effect contributions.

We revised the original sentence "We found that temperature and precipitation seasonality explained 42% of the variation in productivity across plots (Supplementary Table 1)." to "Temperature and precipitation seasonality—the model's fixed effects, or the specific explanatory variables under evaluation—explained 42% of the variation in productivity across plots (Supplementary Table 2, marginal R^2 (R^2_M)=0.42). The full model, which also accounted for random effect (i.e., the variation attributable to the grouping structure of the data, such as quadrats within plots), explained 55% of this variation (Supplementary Table 2, conditional R^2 (R^2_c)=0.55)." (Lines 99-104)

Line 122: The results suggest that the direct effects are not significant. Although I'm

aware that the common use of statistical significance is problematic, I wonder if the quantitative difference between direct and indirect effects isn't worth discussing further.

Thank you for your insightful comment regarding the quantitative difference between direct and indirect effects of climate on forest productivity. We agree that the quantitative difference and significance of direct versus indirect effects are critical for discussion. However, as highlighted in the next paragraph of the Discussion section, the interdependence between direct and indirect pathways creates challenges in attributing causality and accurately quantifying their respective contributions. This interdependence can lead to spurious associations in statistical modeling, potentially overestimating the magnitude of indirect effects. In response to your comment, we have revised our discussion to clarify that while direct effects of climate on productivity are well-established and significant in the literature, our study emphasizes the previously underappreciated indirect pathway via canopy structural diversity. We hope this revision addresses your concern while maintaining the focus of our study on the indirect mechanisms of climate.

We have revised our discussion to clarify this issue:

(In the following lines, we focus on the indirect mechanisms linked to climate:)

"We were able to go beyond this direct effect, however, and found strong support for our first prediction that climate indirectly affects productivity across biomes by altering canopy structural diversity (e.g. crown complementarity, three-dimensional spatial heterogeneity). This mechanistic pathway is not represented in many dynamic global vegetation models, which characterize forest canopies using large-scale mean structural metrics (e.g., leaf area index and height) without accounting for structural diversity. In our study, across broad latitudinal and climatic gradients, covariation between climate and species richness appears to shape structural diversity via physiological constraints on tree height, canopy cover, and vertical complexity. In warm and wet tropical climates, milder hydraulic constraints facilitate taller and multilayered canopies with dense vertical species packing. This stratification is evident in canopy species adapted to high light environments (e.g., via lower specific leaf area and lower leaf phosphorus content) versus understory species specialized for shade (e.g., via higher specific leaf area and leaf phosphorus content). In contrast, colder or drier high-latitude climates impose stronger hydraulic and energy-balance limitations, favoring shorter, less vertically stratified canopies with reduced light partitioning; here, temperate communities tend to exhibit traits favoring hydraulic safety over light capture. Such latitudinal variation in

vertical complexity and niche availability helps explain observed patterns in forest productivity." (Lines 145-163)

6. L.160-164: I agree with the statement about the potential error in the magnitude of indirect pathways, but the statement that the qualitative conclusion is still valid sounds a bit sloppy. Is there anything that can be used to support this statement?

We appreciate your concern that the original phrasing may appear imprecise. In response, we have revised the paragraph to provide stronger justification for the qualitative conclusion by highlighting that the observed patterns of climate-driven structural diversity influencing productivity are consistent with established ecological mechanisms (e.g., hydraulic constraints and light partitioning) and supported by emerging evidence in the literature. We also rephrased the statement to emphasize the robustness of the qualitative relationship, despite uncertainties in the precise magnitude of indirect effects. We hope this revision addresses your concern and provides a clearer and more substantiated argument for the validity of the qualitative conclusion.

We have revised the former discussion as:

"Estimating the indirect effect of climate on productivity is challenging because climate simultaneously exerts direct physiological influences on growth and shapes canopy architecture, creating strong dependence between the "treatment" and the mediator. In cross-sectional structural equation models, this dependence can inflate the estimated mediation pathway, causing some of the direct effects of climate to be misattributed to structural diversity. Nevertheless, while the *magnitude* of indirect effects may be overstated, the qualitative validation of structural mediation is supported by multiple lines of evidence: 1) experimental evidence showing structural complexity enhancements boost productivity independently of large-scale climate variation; 2) consistent covariation of structural complexity and productivity along latitudinal climatic gradients in our analysis and prior work. To resolve this attribution challenge, future work could integrate explicit mechanistic frameworks that disentangle direct climatic drivers from indirect canopy structure-mediated effects, thereby refining the causal interpretation of these relationships." (Lines 172-186)

7. L. 189: Asymmetric competition between shade-tolerant understory and light-demanding canopy species? Maybe I am not getting your point here, but I would expect that competition for light would become less asymmetric and more symmetric

in a situation where more light passes through the canopy and reaches the understorey.
We appreciate your insight regarding the nature of light competition in structurally complex forests. Our intention was to suggest that in highly stratified forests, light acquisition remains strongly asymmetric due to the dominance of tall, light-demanding canopy species that preempt light resources, limiting understory productivity. Even in vertically complex forests, the canopy often intercepts most of the light, maintaining strong competitive asymmetry. We have revised the sentence for clarity as:

"This context dependency likely reflects both intensified asymmetric competition ~~between shade-tolerant understory taxa and light-demanding canopy dominants~~ in vertically complex forests—where canopy dominants suppress understory taxa through light limitation." (Lines 206-208)

8. L. 202: Forest resilience? Why?
We thank the reviewer for highlighting the scope for concern regarding 'forest resilience.' As suggested, we have removed 'Forest resilience' here since our study primarily quantifies climate-structure-productivity relationships rather than resilience mechanisms. The revised text now focuses exclusively on carbon sequestration projections, which aligns with our core findings on the role of structural diversity in mediating climate impacts on productivity.

9. L. 234: Does the number of unidentified taxa differ between biomes, and if so, does this affect the results?
We appreciate this important methodological consideration. Consistent with global biodiversity patterns, tropical plots contained more unidentified taxa. However, their biomass contribution was very little due to biomass dominance by large trees: Following Lutz et al. (2018, Global Ecology and Biogeography) and similar studies (e.g. Stephenson et al. 2014), >50% of plot biomass resided in the largest 1% of trees (all identified to species). Unidentified species were rare trees, contributing very little to total biomass. Therefore, more unidentified taxa in tropical forests does not affect the results.

Lutz, et al. 2018. Global importance of large-diameter trees. Global Ecology and Biogeography 27:849-864.

Stephenson et al. 2014. Rate of tree carbon accumulation increases continuously with

tree size. Nature 507:90-93.

10. Fig. 3: The use of the PCA scores of the plots as a proxy for the climate variable is not very clear (to me). Does this ordinal variable meet the requirements for using this linear model?

We appreciate the reviewer's query regarding our climate PCA approach. We clarify that:

1) PCA scores are continuous variables, not ordinal. They represent weighted linear combinations of original climate variables (MAT, MAP, etc.) along orthogonal axes. For example:

PC1 (71.5% variance) = 0.48*MAP + 0.42*AI + 0.44*MAP-PE-0.32*MAPcv +0.41*MAT_grow +0.37*SR

(MAP, mean annual precipitation; AI, aridity index; MAP-PET, mean annual precipitation minus potential evapotranspiration; MAPcv, precipitation seasonality; MAT_grow, mean temperature in growing season; SR, solar radiation)

2) Linear model assumptions were validated:

Linearity: linear regression plot in Fig. 3f confirmed linear relationships;

3) This approach is standard for handling multicollinear climate variables (e.g., Dormann et al. 2013 Ecography). The PCA axes meet interval-scale requirements for linear models as they are continuous latent variables representing climate gradients.

Dormann, et al. 2013. Collinearity: a review of methods to deal with it and a simulation study evaluating their performance. Ecography 36:27-46.

11. I know that a piecewise SEM requires more data than a normal SEM, but I do not know how many data are needed. Can you give some information?

For piecewise structural equation modeling (piecewise SEM), the required sample size depends on the complexity of the model and the number of parameters being estimated. While traditional SEM typically requires at least 5-10 samples per estimated parameter (Wolf et al. 2013), piecewise structural equation modeling has more random effect variables than traditional structural equation modeling and requires more parameters. On the other hand, piecewise SEM, being a series of linear models, can be more flexible with sample size. Generally, sample sizes of 100-200 are often considered a minimum for stable estimates, with larger samples (e.g., 200+) being beneficial for more complex

models (Shipley 2016). In this study, we have 16 fixed effect variables and 80 random effect variables, with 1568 samples (quadrats) at the 50 m × 50 m scale and 385 quadrats at the 100 m × 100 m scale, thus, the sample size is sufficient for robust parameter estimation in our piecewise structural equation modeling. We also showed the quadrat numbers of 50 m × 50 m and 100 m × 100 m scales in Supplementary Table 1.

Wolf, E. J., K. M. Harrington, S. L. Clark, and M. W. Miller. 2013. Sample Size Requirements for Structural Equation Models: An Evaluation of Power, Bias, and Solution Propriety. Educational and Psychological Measurement 73:913-934.

Shipley, B., 2016. Cause and correlation in biology: A user's guide to path analysis, structural equations and causal inference with R. Cambridge university press.

Reviewer #2 (Remarks on code availability):

Line 14 produces a small error because data1 contains only 20 columns instead of 32.

It would be nice to add some code to show that you have tested the conditions of your statistical models, e.g. normality of residuals for GLM or a Shipley's test of directed separation for SEM (if that makes sense).

The coding issue on Line 14 has been corrected in the revised version of our data and code.

We thank the reviewer for this helpful suggestion. We fully agree that testing model conditions is crucial for robust statistical inference. In our analysis, the response variable (forest productivity) was log-transformed to improve the normality of residuals and homogeneity of variance. To further verify the model assumptions, we examined the distribution of the normalized residuals from the generalized least squares (GLS) models using both visual (Q–Q plots) and statistical (Shapiro–Wilk test) diagnostics. The corresponding R code has been added in Lines 163-172.

In addition, following the reviewer’s suggestion, we performed Shipley's test of directed separation using the dSep() function in the ‘piecewiseSEM’ package (Lines 210, 238). The results confirmed that all conditional independence assumptions were met, indicating that the model structure was appropriately specified and that no important pathways were omitted.

Reviewer #3 (Remarks to the Author):

The authors combined forest inventory data used to estimate biomass (ground truth) with Lidar derived canopy attributes (canopy height, cover and vertical heterogeneity) and worldclim2 database derived climate variables to model productivity changes as a function of plot-level species richness, species diversity (beta diversity), canopy attributes and climate. Authors reported a stronger relation between canopy structural attributes and productivity compared to direct climate-productivity relationship. I find the work interesting but I wonder the novelty in this work. The relationship between stand attributes and productivity has been well published. I understand that authors are taking a larger scale (Biome) approach but I'm not sure there is much new insights from this study.

We thank the reviewer for this important comment, which allows us to clarify the novel conceptual and mechanistic contribution of our study. We agree that positive *correlations* between stand structural diversity and productivity are well-established at local and regional scales. However, our work tried to move beyond documenting this correlation to **quantify the mediating role of canopy structural diversity within the climate-productivity relationship across a global biome gradient**—a mechanism that is critically underrepresented in many ecosystem models.

The novelty of our study may lie in three key aspects:

1) **Shifting from Correlation to Mediation:** While previous studies have shown that structural diversity and productivity are correlated, we employed structural equation modeling (SEM) across a unique, inter-calibrated biome-scale dataset to *partition the effects* of climate. We demonstrate that a significant portion of climate's influence on productivity is not direct, but is *indirectly pathway through* its shaping of canopy structural diversity (Fig. 4) although its magnitude is challenging to isolate from the direct effect of canopy structural diversity. This explicit quantification of the mediation pathway is a key advance.

2) **Resolving a Biome-Scale Mechanistic Pathway:** The reviewer correctly notes we use a multi-biome approach. Its novelty, however, is not just the scale, but the mechanistic insight. By spanning boreal to tropical forests, we reveal that the *strength of the mediation effect itself* is climate-dependent, intensifying toward the tropics. This provides a mechanistic explanation for the latitudinal productivity gradient that integrates both direct physiological constraints and indirect architectural controls.

3) **Addressing a Critical Gap in Ecosystem Models:** As we note in the Discussion (Lines 147-152), many dynamic global vegetation models (DGVMs) represent forest canopies with large-scale *mean* metrics like leaf area index and height. They do not mechanistically incorporate **canopy structural diversity as a dynamic mediator** of climate impacts. Our findings provide the empirical evidence and a quantitative framework necessary for the next generation of models to include this crucial pathway, thereby improving forecasts of carbon sequestration.

In summary, our study does not simply reaffirm a known correlation. It may provide a biome-scale *causal framework* that positions canopy structural diversity as a mediator, explaining *how* climate and diversity jointly influence forest productivity. Please find the following point-to-point responses to your other concerns.

Below are a few additional comments;

1. The data involved in this study occur at different spatial scales; biomass was computed at plot-level (13 to 50 ha plots), the Lidar data has a resolution of 50m x 50m, the climate data had a resolution of 1 km2. Authors did not explain how they dealt with the differences in scale. Were the inventory plots spaced out 1 km apart to match the climate data?

We thank the reviewer for highlighting this important methodological consideration. We clarify that:

1） Biomass and canopy structural metrics (i.e., height, cover and complexity) were computed at matching spatial scales—50m × 50m and 100m × 100m quadrat resolutions although plot size is in the range 15.32-50 ha (See supplementary Table 1; Lines 73-77, 253-258);

2） Climate is typically assumed to be homogeneous within each 1 km^2 grid cell, with forest plots fully contained within individual grid cells and centered accordingly. Microclimatic variation within each plot may be partially explained by local topographic variables, but this is not considered.

3） Minimum inter-plot distance exceeds 100 km (Supplementary Fig. 1a, Supplementary Table 1), preventing spatial autocorrelation.

We hope these explanation addresses your methodological concerns regarding on the issue of scale.

Authors did not mention how old these forest stands are at the census time (2010-2019). This is important to understand how relevant the 1971-2000 climate data/reference is regarding biomass accumulation.

Thank you for your thoughtful comment regarding the temporal alignment between the climate data (1971–2000) and biomass accumulation (2010–2019), as well as your inquiry about the age of the forest stands. We acknowledge the importance of stand age for biomass accumulation. As all 16 forest plots are natural mature forests "Plots were drawn from the ForestGEO and the Chinese Forest Biodiversity Monitoring networks and represent natural forests with minimal anthropogenic disturbance." (Lines 235-237 in Methods section). Moreover, we also added mean individual basal area to as a proxy of development stage, skewness of DBH (diameter at breast height) class distribution using 5 cm interval (where a positive skew indicates a regenerating stand) and net annual biomass accumulation (where high net increases suggest recovery and net decreases signal recent disturbance) to account for local natural disturbance.

We also acknowledge the importance of clarifying the relationship between the timing of biomass accumulation and the climate data used in our study. Regarding the use of 1971–2000 climate data, our rationale is based on two main considerations:

1) **Climatic Baseline Representation:** The 30-year mean climate (1971–2000) represents a standard climatological baseline that captures long-term average conditions and reduces the influence of short-term variability (as shown in the following Table). This is a commonly used period in ecological studies and provides a stable reference for comparing spatial differences across regions.
2) **Spatial differences in climate**: While the census period (2010–2019) is more recent, the large climatic variation among our distantly distributed plots ensures that the relative differences in climate (e.g., temperature, precipitation) across plots are well-represented by the 1971–2000 data (as shown in below table). These differences, rather than absolute climate values, are critical for explaining variations in biomass accumulation across our study sites.

For these reasons, WorldClim data are commonly used in the literature to represent long-term average climate. For examples, Ehbrecht et al. (2021) relied on it to examine the drivers of canopy structural complexity, while LaRue et al. (2023) used it as the climatic basis for their analysis of forest productivity derived from Forest

Inventory and Analysis data collected between 2004 and 2019. For the purpose of this study—focusing on spatial variation in forest biomass—the use of long-term mean climate data may provide a reasonable and robust climatic context. We agree that the potential influence of more recent climate trends on biomass accumulation is worth exploring in future studies, especially in the context of climate change.

Climatic variables of 16 forest plots extracted from WorldClim2 database.

Plot name	MAP	AI	MAP-PET	MAPcv	SR	MAT_grow
Danum valley	2346	14785	760	15.92	17415.17	25.58
Paracou	2759	17333	1168	49.96	18275.75	25.65
Barro Colorado Island	2210	13158	531	64.48	19277.17	26.00
Luquillo	2110	12376	406	27.37	18758.50	23.25
Xishuangbanna	1695	11761	254	77.86	16309.58	23.29
Ailaoshan	985	9271	-77	78.44	15109.83	15.26
Mulun	1379	11813	212	68.57	13642.42	18.40
Gutianshan	1788	14943	592	56.58	14595.33	15.40
Tiantong	1358	11675	195	43.36	14308.17	14.71
Utah	568	3871	-899	24.41	17562.25	10.28
Smithsonian Conservation Biology Institute	1026	8306	-209	13.31	13990.25	15.29
Donglingshan	504	3659	-873	109.73	15591.33	13.99
Changbaishan	680	7404	-238	99.22	13505.75	15.02
Wind River	2008	18467	921	65.90	12799.50	12.34
Fenglin	623	6535	-330	105.58	13385.17	15.44
Daxinganling	538	7074	-222	104.88	12163.08	10.78

Key to abbreviations: MAP, mean annual precipitation; AI, aridity index; MAP-PET, mean annual precipitation minus potential evapotranspiration; MAPcv, precipitation seasonality; MAT_grow, mean temperature in growing season; SR, solar radiation

3. Authors mentioned that there were 16-forest dynamics plots but the Figure 2 data has several data points. There were also random mentions of quadrat with no explanation how this is connected with plots and the Figure 4 mentioned n=1568 quadrats.

Thank you for pointing out the need for clarification regarding the data representation in Figure 2 and the references to quadrats. We acknowledge that the connection between the 16 forest dynamics plots, the quadrats, and the data presented in Figures 2 & 4 needed better explanation in the original manuscript.

We apologize for the confusion and have revised the manuscript to improve clarity. Specifically:

1) Figure 2 does not display raw data points from the 16 forest dynamics plots. Instead, each point represents a standardized regression coefficient derived from one of six separate mixed-effects linear models (six climate predictors × one response variable) per panel. These coefficients reflect the strength and direction of the relationships at the model level, not individual observations.
2) To achieve robust parameter estimation, these models were based on data from a large number of quadrats within the 16 forest plots. These quadrats, of 50 m × 50 m and 100 m × 100 m in size, served as sampling units for model fitting.
3) As correctly noted, Figure 4 refers to n = 1,568 quadrats, which is the total number of quadrats used from all plots. We have now clarified this in the figure captions and the main text to avoid further confusion (Lines 76, 602).
4) Additionally, we have added a sentence in the Introduction to briefly explain the use of quadrats as spatial sampling units within plots (Lines 76-77), and we now revised Supplementary Table 1, which provides the number and size of quadrats per plot. Moreover, we also explain why we chose quadrat size 50 m × 50 m and 100 m × 100 m as target scales in Method section as following: "Each forest plot was divided into non-overlapping 50 m × 50 m (49-200 per plot) and 100 m × 100 m (6-50 per plot) quadrats to balance the trade-off between minimizing the uncertainty in biomass estimation from border effects and maintaining sufficient sample size for robust cross-plot statistical analysis (Supplementary Table 1). This design mitigates the ~40% uncertainty from

border effects at a 20 m × 20 m scale while ensuring sufficient sample size.” (Lines 253-258)

We hope these revisions resolve the misunderstanding and provide a clearer connection between the forest plots, quadrats, and the figures presented.

4. I was also wondering why authors did not included historical disturbances in their model.

Thanks for your constructive suggestion for inclusion of historical disturbances into our model. Reviewer 1 also had similar suggestion. Following your suggestion, we have expanded our conceptual introduction in the manuscript, added natural disturbance as a driver of species diversity, canopy structure and forest productivity as in Figure 1.

We then used three metrics to approximate natural disturbance history: 1) mean individual basal area per quadrat as a proxy of development stage, 2) skewness of DBH class distribution using 5 cm intervals, where a positive skew indicates a regenerating stand, 3) net annual biomass change, where high net increases suggest recovery and net decreases signal recent disturbance. We selected net annual biomass change as our representative measure because it directly quantifies the functional response of the ecosystem to disturbance by integrating the net effects of growth, recruitment, and mortality on carbon dynamics. The results of the updated models are presented in Figure 3, and the 'Methods' section has been updated accordingly (Lines 352-367). Its inclusion did not qualitatively change our main conclusions but explained a greater portion of the variance in forest productivity (previously 54% vs current 59%), species richness (54% vs 54%) and canopy structure (canopy cover 32% vs 34%, canopy height 43% vs 44%, vertical structural complexity 39% vs 39%) as shown in Figure 4.

5. In the SEM (Figure 4a), which climate variable is represented? authors mentioned that they performed PCA and retained the first principal axis (supplement Table 7) - was any particular climate variable used in the SEM based on the PCA results?

Thank you for your insightful question regarding the climate variable used in the Structural Equation Model (SEM) shown in Figure 4a.

To clarify, in the SEM analysis, **we used the first principal component (PC1) from the PCA of six climate variables** as a composite climate predictor. This approach allowed us to reduce dimensionality while capturing the major gradient in climate variation across the study sites.

As described in Supplementary Table 7, **PC1 explained 71.5% of the total variance** in the original climate variables and represents a weighted linear combination of six climatic variables used in this study: 1) Mean annual precipitation (MAP); 2) Aridity index (AI); 3) Mean annual precipitation minus potential evapotranspiration (MAP-PET); 4) Precipitation seasonality (MAPcv); 5) Mean temperature during the growing season (MAT_grow); 6) Solar radiation (SR).

The equation for PC1 is as follows (coefficients from Supplementary Table 7):

$$PC1 = 0.48 \times MAP + 0.42 \times AI + 0.44 \times MAP\text{-}PET - 0.32 \times MAPcv + 0.41 \times MAT\_grow + 0.37 \times SR$$

This composite axis reflects a combined gradient of water and energy availability, with positive PC1 values indicating more humid and warmer environments.

We have clarified this point in the revised manuscript by specifying that PC1 scores were used as the climate variable in the SEM, and we now explicitly reference the PCA results and loadings (Supplementary Table 7) in the Methods section as following:

"To reduce the number of climatic variables and avoid multicollinearity, we performed a principal component analysis (PCA) and retained the first axis of the PCA (PC1) (71.5% variance explained; Supplementary Table 7) as an integrated representation of the full climate variable set." (Lines 394-398)

We hope this addresses your concern and improves the transparency of our modeling approach.

6. Line 90; reference to supplementary Table 1 here is incorrect.

We revised mistaken references as supplementary Table 2.

Response to reviewer comments:

Reviewer #1 (Remarks to the Author):

I wish to thank the authors for their revisions in response to the previous round of reviews. I believe these revisions have improved the paper. Overall, I remain impressed by the LiDAR and associated forest inventory data, and I find the broad topic of the paper interesting.

We thank you for your positive response to our revision and constructive feedback, which has significantly strengthened our manuscript. We have revised the text with major improvements detailed below. We have

1) Explicitly defined the mechanistic pathways (space filling, light partitioning and vertical niche differentiation) through which canopy structural diversity affects productivity.
2) clearly stated that our key contribution is quantifying how climate modulates the structural diversity–productivity relationship across biomes, not merely documenting the expected correlation.
3) Strengthened the model justification, detailing the consequences of omitting structural diversity in many dynamic global vegetation models.

We have also incorporated all specific line-edits, removed the problematic beta-diversity analysis, clarified methodologies, and confirmed data availability. We hope that these revisions have addressed all your concerns, and our manuscript is more rigorous and better-justified.

However, I found the revised paper remained hard going in places. While the results are quite interesting, I found myself wondering about the direction of causation and whether the observed relationships between canopy structure and productivity are not surprising and would be expected to emerge by definition. This issue aside, my main concerns centre around the conceptual framing, in particular the way structural diversity is presented as a ‘mechanism’. As it is currently presented in the paper, I did not find this convincing and I believe that some further conceptual development is needed. What are the processes by which canopy structure ought to affect productivity (is this about space filling, partitioning of light environments within the canopy,

something else, or all of the above?)? What is the evidence that canopy structure is a driver rather than passenger of productivity? The structural equation model is fine, but I think a deeper process-based explanation is needed; an explanation that draws upon resource partitioning or physiological trade-offs or other processes.

We thank you for these critical insight on conceptual framing and causality, which has significantly improved the conceptual framing of our paper. we have revised the manuscript to:

1) *Reframe canopy structural diversity as plausible mediator of productivity*. Regarding the direction of causation, we agree that distinguishing whether canopy structural diversity acts as a driver or a passenger of productivity is a fundamental issue. To improve conceptual clarity, we have revised the manuscript to reframe canopy structural diversity not as a mechanism itself, but as an emergent canopy property that plausibly mediates productivity by influencing well-established light-related processes at the stand level. Specifically, we now describe how climate-driven variation in structural diversity can indirectly shape forest growth across biomes by modifying space filling, light-environment partitioning, and the potential for vertical niche differentiation among coexisting species with divergent leaf and crown traits. These widely recognized processes provide a process-based framework for interpreting the statistical pathways identified by our model, while making clear that they are not directly tested and that the inferred relationships represent plausible mediating pathways rather than demonstrated causal mechanisms.

2) *clarify that our key contribution*. In response to the concern that the structural diversity–productivity relationship might appear "expected by definition," we now clarify that our key contribution is not simply documenting a positive structural diversity-productivity relationship, but demonstrating—how climate modulates the relationship between structural diversity and productivity across biome-scale gradients. Our structural equation model supports the indirect pathway through which climate shapes productivity via structural diversity, beyond climate's direct physiological effects. This elevates structural diversity from a descriptive correlate to an underappreciated mediator of large-scale productivity patterns.

We have also revised the first paragraph of Introduction to clarify the direction of causation and to better distinguish climate's direct physiological effects from its indirect influences mediated through structural changes.

"Forest canopies—the collective layer of tree crowns in a stand—capture up to 98% of incoming solar energy in dense forests, highlighting the role of canopy structural diversity in driving ecosystem energy capture. The canopy structural diversity–the three-dimensional heterogeneity in volume and arrangement of above-ground vegetation (e.g., leaves and branches)–is an emergent property arising from three components: canopy height, canopy cover, and vertical complexity (i.e., canopy depth and vertical leaf area distribution). These components are expected to influence productivity through three mechanistic pathways: (1) space filling, where greater horizontal cover and vertical stratification increases the total light-intercepting leaf area; (2) light-environment partitioning, where heterogeneous arrangements of leaf area create diverse light niches; and (3) vertical niche differentiation, where species with divergent leaf and crown traits exploit these niches more efficiently. Consequently, ~~These components govern light interception and utilization efficiency~~ structural diversity is hypothesized to governs not only the total amount of light intercepted but also how light is partitioned and utilized within canopies of a stand, thereby directly coupling canopy structural diversity to stand-scale productivity. The strength of this relationship, however, may be modulated by climate via two pathways: directly, through physiological constraints imposed by water stress and temperature extremes on individual tree growth, and indirectly, ~~by altering~~ through climate-driven changes in structural diversity ~~that determines light interception and use efficiency~~. These structural changes may modify the internal light environment, potentially influencing overall light interception and use efficiency by facilitating exploitation among coexisting species with divergent leaf traits or by limiting it in species with less differentiated leaf traits. Resolving the mechanistic link between these climate-driven shifts in structural diversity and observed productivity patterns across spatial gradient thus requires disentangling the dual roles of climate: its direct physiological constraints on growth versus its indirect effects on productivity mediated by structural diversity." (Line 19-48)

Similarly, we revised the second paragraph of the Introduction following the conceptual framework (See details between Lines 49-76). Accordingly, we revised the first paragraph of Discussion as following (Lines 151-191):

"We were able to go beyond this direct effect, however, and found strong support for our first prediction that climate indirectly affects productivity across biomes by altering canopy structural diversity (e.g. crown complementarity, three-dimensional spatial heterogeneity). Structural diversity governs energy capture processes—such as space filling and light environment partitioning that enable vertical niche differentiation—that regulate how efficiently stands convert light into biomass. While previous research has emphasized species diversity as a driver of productivity via structural diversity at the community level, our findings highlight that climate-driven variation in structural diversity itself may play an underappreciated role in modulating productivity across biomes. ~~This mechanistic pathway is not represented in many dynamic global vegetation models, which characterize forest canopies using large-scale mean structural metrics (e.g., leaf area index and height) without accounting for structural diversity.~~ In our study, across broad latitudinal and climatic gradients, covariation between climate and species richness appears to shape structural diversity via physiological constraints on tree height, canopy cover, and vertical complexity. In warm and wet tropical climates, milder hydraulic constraints facilitate taller, and multilayered canopies with dense vertical species packing. This stratification is evident in canopy species adapted to high light environments (e.g., via lower specific leaf area ~~and lower leaf phosphorus content~~) versus understory species specialized for shade (e.g., via higher specific leaf area ~~and leaf phosphorus content~~). In contrast, colder or drier high-latitude climates impose stronger hydraulic and energy-balance limitations, favoring shorter, less vertically stratified canopies with reduced light partitioning. These climate-dependent shifts in structural diversity have direct consequences for stand-level productivity: more stratified canopies capture and utilize light more efficiently, whereas simplified canopies limit stand productivity. ~~Such latitudinal variation in vertical complexity and niche availability helps explain observed patterns in forest productivity. While previous research has primarily emphasized the role of species diversity in driving productivity via canopy structural diversity at the community level, our findings highlight that climate-driven variation in structural diversity itself may play an underappreciated role.~~ A key implication is

that structural diversity is missing from many dynamic global vegetation models, which characterize forest canopies using large-scale mean structural metrics (e.g., leaf area index and height) and therefore cannot capture structural diversity that governs those processes. This omission may bias productivity estimates-underestimating productivity in structurally complex tropical forests while overestimating it in simpler high-latitude forests. Integrating climate-structural diversity-productivity feedbacks into next-generation ~~dynamic global~~ vegetation models~~—moving beyond mean structural metrics toward more mechanistic representations of structural diversity—~~ will improve ecosystem service~~s~~ projections and inform climate-adaptive forest management." (Lines 151-191)

We hope these changes have addressed your concerns and improve the conceptual clarity and motivation of our study.

Related, a key message in the paper is that canopy structure needs to be considered in ecosystem models. I do not necessarily disagree. But the reasons *why* this complexity is needed are not clearly explained. To justify adding this kind of complexity in models, there ought to be a clear process-based pathway by which canopy structure represents a dynamic with meaningful consequences – alongside evidence of the consequence of not representing this dynamic within models. To reiterate, I think there probably is justification, I just do not think this is currently clearly articulated.

Thank you for this thoughtful and constructive comment for clearer justification. Regarding the need to represent canopy structure in ecosystem models, we have revised our manuscript to articulate why neglecting these processes in models can affect predictions of productivity and ecosystem services. Specifically, we now argue that because space filling and light-environment partitioning directly affect light interception and use efficiency—core controls on photosynthesis—models that rely only on mean structural metrics (e.g., LAI and canopy height) may miss climate-driven variation in structural diversity with meaningful consequences for productivity.

We further highlight that omitting structural diversity may lead to systematic biases, such as underestimating productivity in structurally complex tropical forests and overestimating it in simpler, high-latitude forests. By explicitly linking canopy structure to light capture processes and to potential model biases, we clarify why incorporating structural diversity represents a dynamic with tangible consequences for ecosystem projections as following:

"A key implication is that structural diversity is missing from many dynamic global vegetation models, which characterize forest canopies using large-scale mean structural metrics (e.g., leaf area index and height) and therefore cannot capture structural diversity that governs those processes. This omission may bias productivity estimates-underestimating productivity in structurally complex tropical forests while overestimating it in simpler high-latitude forests. Integrating climate-structural diversity-productivity feedbacks into next-generation dynamic global vegetation models~~—moving beyond mean structural metrics toward more mechanistic representations of structural diversity—~~will improve~~ risk assessment for~~ ecosystem service~~s~~ projections and inform climate-adaptive forest management." (Lines 183-191)

We hope these revisions have addressed your concerns.

Some specific comments:

Introduction: I found the reasoning somewhat unclear or lacking precision in places. For example, on line 28, 'physiological constraints' is broad and somewhat unclear; could the authors explain what they mean with a little more precision?

We thank you for this helpful suggestion. To provide greater precision, we have revised the sentence to specify the key physiological processes involved, namely constraints related to water stress, temperature extremes, shortened growing season, reduced photosynthetic capacity. We revised former sentence as "The strength of this relationship, however, may be modulated by climate via two pathways: directly, through physiological constraints imposed by water stress and temperature extremes on individual tree growth, …" (Lines 36-39)

Similarly, on line 45, the ‘synergy between climate effects’ and what? And, on line 49, what is meant by ‘leaf types’? Is this referring to species that have different photosynthetic responses to light?

(1) We agree and thank you for highlighting this lack of clarity. on line 49, the intended meaning was the synergy between the *direct and indirect climatic effects* described in the previous sentence. We have revised the text to explicitly state this, thereby improving precision and logical flow.

“The synergy between climate-driven shifts in structural diversity and direct physiological effects intensifies toward the tropics, ~~which~~ thereby strengthening the relationship between structural diversity and forest productivity along latitudinal gradients.” (Lines 60-63)

(2) Yes, ‘leaf types’ refers to species that have different photosynthetic responses to light. By "leaf types", we refer to variation in key leaf functional traits—such as leaf mass per area (LMA) and nitrogen content—that directly govern differential photosynthetic responses to light. In the revised Introduction, we have now explicitly detailed the mechanistic pathways by which structural diversity influences productivity. Accordingly, we have removed this redundant sentence in question. We hope this revision directly addresses your concern.

Lines 29-30: This implies that canopy structure generates light use efficiency. It would be helpful to more clearly spell out the role of physiological differences among trees.

We agree that the original phrasing did not explicitly connect the alteration of structural diversity to the physiological mechanisms of exploitation. We have revised the sentence to clarify that alteration of structural diversity modifies light environment, which in turn can enable or limit physiological adaptations (e.g., to high or low light), thereby influencing overall light use efficiency. We revise former sentence as:

"The strength of this relationship, however, may be modulated by climate via two pathways: directly, through physiological constraints imposed by water stress and temperature extremes on individual tree growth, and indirectly, ~~by altering~~ through changes in structural diversity ~~that determines light interception and use efficiency~~. These structural changes modify the internal light environment, potentially influencing overall light interception and use efficiency by facilitating exploitation among coexisting species with divergent leaf traits or by limiting it in species with less differentiated leaf traits." (Lines 36-44)

Line 83-85: This biological/ecological interpretation of beta-diversity is not clear to me. This suggests that beta-diversity will, in itself, give insight into the dominance of fast-growing species. I do not see how this is possible. Could this be revised and clarified?

We thank you for this important critique, which helped us clarify a conceptual overreach in our initial framing. Our original hypothesis was that localized disturbance could create quadrats with distinct species compositions (high beta-diversity) dominated by fast-growing pioneers, thereby influencing productivity. However, as beta-diversity is primarily driven by environmental heterogeneity, dispersal limitations, and the regional species pool in natural forests, and thus has minimal direct influence on productivity across plots as shown in our analyses. This aligns with the synthesis of van der Plas et al. (2023), who found no consistent universal relationship between beta-diversity and ecosystem functioning. As a result, we have removed beta-diversity from our analysis to avoid confusion and ensure a more accurate representation of the drivers of productivity. We hope we have addressed your concerns regarding the effect of beta-diversity on productivity.

van der Plas, F., J. Hennecke, J. M. Chase, J. van Ruijven, and K. E. Barry. 2023. Universal beta-diversity–functioning relationships are neither observed nor expected. Trends in Ecology & Evolution 38:532-544

Line 96-97: Why test both aridity index (P/PET) and water balance (P - PET)? Unsurprisingly they are very tightly correlated (Supplementary Table 6) and have a similar interpretation. Unless there is a clear reason that I am missing, I would be inclined to choose and show one (probably the aridity index).

Thank you for this important methodological comment. As you pointed out, the aridity index (P/PET) and water balance (P − PET) are highly correlated ($r = 0.98$), reflecting their close conceptual relationship in characterizing water deficit. While the aridity index is widely used to represent long-term climatic dryness, water balance more directly reflects short-term variability in water availability.

We conducted a sensitivity analysis and found that the correlation between PC axis 1 of climate and vertical structural complexity (quantified as foliage height diversity) shifted from significant ($p=0.0496$) to marginal significant ($p=0.0599$) after excluding water balance from PCA (principal component analysis) for our structural equation modeling. However, the effect size (standardized coefficients) of PC axis 1 of climate remained nearly unchanged (0.4083 vs 0.4058). We thus exclude water balance from our analysis.

Line 124: Would this be expected to emerge by definition (i.e., given the way that disturbance has been characterised)?

We agree that the positive correlation between natural disturbance and productivity may appear definitional depending on how disturbance is characterized. In our manuscript, natural disturbance was not intended to be defined in a way that presupposes this relationship. To clarify this, we have now (1) added a concise definition of natural disturbance before the Results section, emphasizing that it is an independently quantified variable as "Topographic variables (e.g., elevation and slope) and quadrat-scale natural disturbance (i.e., events caused by non-anthropogenic agents—such as fire, wind, or pathogens—that significantly alter forest structure and functions, initiating succession, see more details in 'Methods') were included as covariates to account for local environmental conditions and natural disturbance." (Lines 101-106)

And (2) revised the sentence in the Results section to report the statistical outcome without implying causation or expectation. “In contrast, natural disturbance ~~was consistently positively correlated~~ exhibited a consistent positive correlation with productivity.” (Lines 136-138)

We hope this resolves the potential ambiguity.

Line 279: As flagged above, I do not follow this interpretation of beta-diversity (beta-diversity is a metric of species turnover but does not reveal the identity or functional characteristics of species).

As we responded to your above comments, we have removed beta-diversity from our analysis to avoid confusion and ensure a more accurate representation of the drivers of productivity. We hope this issue does exist.

Line 252: Am I correct in understanding that trees reaching the threshold size to be included in the census for the first time had their entire biomass considered as new growth? This would seem to overestimate biomass growth.

We thank you for this methodological question. You are correct in your understanding that trees reaching the minimum census threshold (DBH ≥ 10 cm) for the first time have their full biomass included as part of the "new recruit" component for that census interval. This is a standard practice in many forest census studies and, as you noted, it results in an initial overestimation of aboveground productivity for that specific tree during the census interval. However, this potential bias is minimized and highly unlikely to affect the overall conclusions for the following two reasons in our study for two reasons:

1) Dominance of large trees: As supported by Chisholm et al. (2013), trees ≥ 10 cm DBH constitute 91.3 ± 3.8% (mean ± standard deviation) of the aboveground productivity of all trees ≥ 1 cm DBH.
2) Compensating Errors: As you correctly point out, the initial inclusion of a recruit's full biomass overestimates productivity. However, this is largely cancelled out by an underestimation of productivity. Specifically, the biomass growth of trees that were initially < 10 cm DBH in the first census and are still < 10 cm DBH in the

second census is not included in our calculation. These trees are growing, but their growth is not captured because they have not yet crossed the minimum threshold. This uncaptured growth (underestimation) effectively mitigates the impact of the full-biomass inclusion of new recruits (overestimation).

We hope this clarifies the approach and its implications for our analysis.

I may have missed it, but I could not see the data availability statement. Will the LiDAR data be made available?

Thank you for pointing out the apparent omission of the data availability statement. Regarding the Data Availability Statement: You did not miss the statement; it was temporarily placed in the Cover Letter (and not in the main manuscript text) to maintain double-blind review integrity.

Regarding Data Availability: We confirm that the core data used in this study has been made publicly available. The following comprehensive dataset is currently deposited in the Zenodo repository and Code Ocean.

1) The components of canopy structural diversity derived from LiDAR data, including canopy cover, height, and vertical structural complexity for each 50 m × 50 m quadrat.
2) Ecological variables for each quadrat, including aboveground productivity, proxies of natural disturbance, species richness and β-diversity.
3) Climatic variables for each plot and topographic variables for each quadrat.

Reviewer #2 (Remarks to the Author):

Dear authors, thank you very much for carefully revising your manuscript. I appreciate the way you have addressed the comments and the effort you have made to clarify unclear sentences or content. I confirm that you have addressed all my comments in sufficient detail.

Reviewer #2 (Remarks on code availability):

I have also reviewed the R code you provided via Code Ocean. It now works flawlessly and is very helpful for understanding the methodology. I have no further comments.

We thank Reviewer #2 for their positive final assessment and validating the reproducibility of our R code.

Reviewer #3 (Remarks to the Author):

Authors addressed my earlier comments quite satisfactorily. I do think the subject is not excessively novel (see these two papers below), however, authors argue that dynamic global vegetation models (DGVMs) represent forest canopies with large-scale mean metrics like leaf area index and height so this work can change that by emphasizing the need to incorporate canopy structural diversity - is a good response.

Paquette, A. and Messier, C., 2011. The effect of biodiversity on tree productivity: from temperate to boreal forests. Global Ecology and Biogeography, 20(1), pp.170-180.

Anyomi, K.A., Raulier, F., Bergeron, Y. and Mailly, D., 2013. The predominance of stand composition and structure over direct climatic and site effects in explaining aspen (Populus tremuloides Michaux) site index within boreal and temperate forests of western Quebec, Canada. Forest ecology and management, 302, pp.390-403.

We thank Reviewer #3 for the positive assessment of our revisions and for providing the two relevant references (Paquette Messier 2011, Anyomi et al. 2013). We appreciate the opportunity to clarify the novel aspects of our work in relation to these studies.

While the cited papers valuably explore the relationship between species richness/composition and forest productivity, our study provides a distinct and complementary contribution by focusing explicitly on canopy structural diversity as a key mediator. Specifically, our work offers novelty in three interconnected ways:

1) *Mechanistic Focus on Structural Diversity*: We directly quantify how climate shapes LiDAR-derived canopy structural diversity (height, cover, and vertical complexity) across biomes. We then test how this structural diversity, after accounting for the effect of species richness, mechanistically influences productivity through processes like light partitioning and niche differentiation.
2) *Empirical Evidence for a Biome-Scale Pathway*: We provide cross-biome empirical evidence for a consistent climate→structural diversity→productivity pathway. This demonstrates that climate's indirect effect, mediated by structural diversity, is a significant and generalizable driver complementing its direct physiological effects.

This finding together with 1) provides distinct and complementary contribution compared with those of the two cited papers.

3) *Direct Implications for Model Development*: A central conclusion is that current Dynamic Global Vegetation Models (DGVMs), which rely on mean structural metrics, fail to capture this pathway. We explicitly articulate the consequence of this omission: the potential for systematic biases in productivity estimates (e.g., underestimation in complex tropical forests and overestimation in simpler boreal systems), which has clear implications for improving carbon cycle projections.

In line with your comment, we have cited the two suggested papers (Paquette Messier 2011, Anyomi et al. 2013) in the revised manuscript to better contextualize our study within the existing literatures.
